# Abscisic acid regulates stomatal production by imprinting a SnRK2 kinase–mediated phosphocode on the master regulator SPEECHLESS

**DOI:** 10.1126/sciadv.add2063

**Published:** 2022-10-07

**Authors:** Xin Yang, Lalitha Gavya S, Zimin Zhou, Daisuke Urano, On Sun Lau

**Affiliations:** ^1^ Department of Biological Sciences, National University of Singapore, 14 Science Drive 4, Singapore 117557, Singapore.; ^2^ Temasek Life Sciences Laboratory, 1 Research Link, Singapore 117604, Singapore.

## Abstract

Stomata, the epidermal pores for gas exchange between plants and the atmosphere, are the major sites of water loss. During water shortage, plants limit the formation of new stoma via the phytohormone abscisic acid (ABA) to conserve water. However, how ABA suppresses stomatal production is largely unknown. Here, we demonstrate that three core SnRK2 kinases of ABA signaling inhibit the initiation and proliferation of the stomatal precursors in *Arabidopsis*. We show that the SnRK2s function within the precursors and directly phosphorylate SPEECHLESS (SPCH), the master transcription factor for stomatal initiation. We identify specific SPCH residues targeted by the SnRK2s, which mediate the ABA/drought-induced suppression of SPCH and stomatal production. This SnRK2-specific SPCH phosphocode connects stomatal development with ABA/drought signals and enables the independent control of this key water conservation response. Our work also highlights how distinct signaling activities can be specifically encoded on a master regulator to modulate developmental plasticity.

## Teaser

The drought stress hormone limits stomatal formation by destabilizing the master regulator SPEECHLESS via a specific phosphocode.

## INTRODUCTION

Water is a limited resource, and land organisms, especially sessile ones like plants, must adapt to periodic water shortages. In plants, most absorbed water is lost via transpiration—the release of water vapor through the microscopic pores called stomata (*1*). During drought, the phytohormone abscisic acid (ABA), which controls water-use efficiency and drought tolerance, accumulates, and it restricts transpiration loss by initiating two stomatal responses: the closing of the stomatal pores and the suppression of stomatal formation (*2*–*6*). The complementary responses, operating at different time scales, allow plants to balance carbon uptake and water loss in changing water conditions.

The core ABA signaling module is composed of ABA receptors, clade A protein phosphatases type 2C (PP2Cs), and the three subclass III SNF1-related protein kinases (SnRK2s): SnRK2.2/SRK2D, SnRK2.3/SRK2I, and SnRK2.6/SRK2E/OPEN STOMATA 1 (OST1) (*7*–*10*). In the presence of ABA, the ABA-bound receptors inhibit PP2Cs, relieving PP2C’s suppression of the SnRK2s. The ABA-activated SnRK2s (*4*, *11*) then phosphorylate downstream targets to mediate ABA-induced responses. In stomatal guard cells, several ion channels, such as SLOW ANION CHANNEL 1 (SLAC1), and transcription factors are well-established direct targets of SnRK2.6, and SLAC1 phosphorylation results in stomatal closure (*3*, *12*–*15*). However, how ABA inhibits the production of stomata and coordinates the two cell type–specific responses remain unclear.

In flowering plants, a specialized epidermal cell lineage is responsible for generating guard cells (*16*–*18*). Similar to adult stem cell lineages in animals, the stomatal lineage consists of several distinct cell stages, and precursors of the lineage, i.e., meristemoid mother cells (MMCs) and meristemoids, have self-renewing ability, allowing plasticity in stomatal production. The specification and proliferation of the stomatal precursors relies on the master regulator SPEECHLESS (SPCH), a basic helix-loop-helix (bHLH) transcription factor, which regulates cell fate by controlling hundreds of genes (*19*, *20*). SPCH also acts as a major node in the modulation of stomatal production by several intrinsic and environmental factors, such as brassinosteroid, light, and warmth, and is regulated transcriptionally and posttranslationally by these pathways (*21*–*28*). Since mutants with low ABA levels displayed prolonged expression of *SPCH* (*6*), it is possible that ABA signaling influences *SPCH* level/activity in restricting stomatal production. Here, we demonstrate that the three subclass III SnRK2s mediate the ABA-dependent suppression of stomatal production early in their development, that they phosphorylate SPCH at specific residues in vitro and in vivo, and that the SnRK2-specific phosphocode transduces signals from ABA, drought, and likely osmotic stress, in suppressing SPCH and stomatal production in Arabidopsis.

## RESULTS

### The three subclass III *SnRK2*s are required for the ABA-mediated suppression of stomatal development

**Fig. 1.**
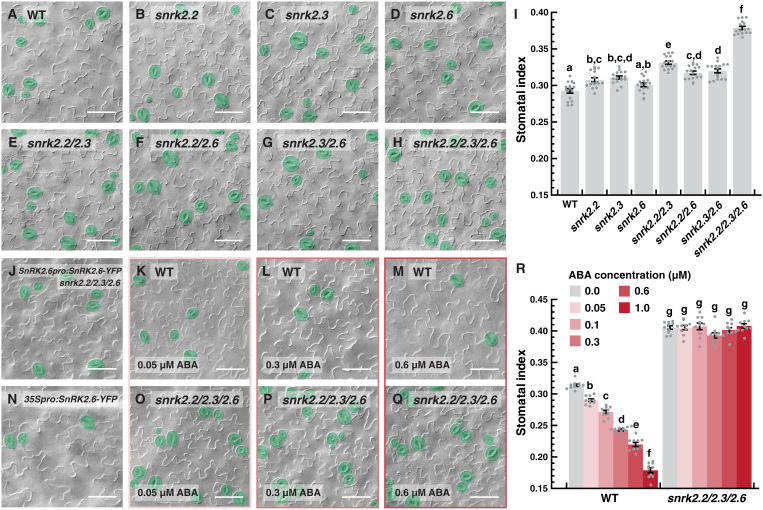
The three subclass III *SnRK2*s mediate ABA-dependent suppression of stomatal development. (**A** to **I**) Representative images (A to H) and quantification of stomatal indices (I) of 10-day-old abaxial cotyledons of wild-type (WT; A) and single (B to D), double (E to G), and triple (H) mutants of the three subclass III *SnRK2* kinases, which include *SnRK2.2/SRK2D*, SnRK2*.3/SRK2I*, and *SnRK2.6/SRK2E/OST1*. SI refers to the ratio of stomata to total epidermal cells. (**J** and **N**) Representative images of 10-day-old abaxial cotyledons of the *snrk2* triple mutant complemented by *SnRK2.6pro:SnRK2.6-YFP* (J) and an overexpression line of *SnRK2.6*, *35Spro:SnRK2.6-YFP* (N). Quantifications of their stomatal indices can be found in fig. S1. (K to M and **O** to **R**) Effect of ABA on stomatal development in WT and the *snrk2* triple mutant. Seedlings were grown for 3 days and transferred to ABA-containing medium at the indicated concentrations for seven more days before examination. Representative images of select cotyledons (K to M and O to Q) and stomatal indices (R) are shown. For (A) to (H) and (J) to (Q), stomata are pseudo-colored in green. Scale bar, 50 μm. Values are means ± SEM; *n* = 16 (I) or 10 (R) independent cotyledons. One-way (I) or two-way (R) analysis of variance (ANOVA) with Tukey’s multiple comparisons test, *P* < 0.05.

Mutant analyses have demonstrated the importance of ABA metabolism and perception in the regulation of stomatal development (*5*, *6*, *29*). To dissect how downstream ABA signaling is involved, we focused on the three SnRK2 kinases. A previous study reported that the suppression of stomatal number by ABA is independent of *SnRK2.6* (*30*). Since only *snrk2.6* mutants were tested, gene redundancy within the subfamily may explain the lack of phenotype. Therefore, we comprehensively examined stomatal production in wild type (WT), the three single loss-of-function *snrk2* mutants (i.e., *snrk2.2*, *snrk2.3*, and *snrk2.6*), the three combinations of double *snrk2* mutants, and the triple *snrk2* mutants (Fig. 1, A to I). Stomatal production was scored on abaxial cotyledons of 10-day-old seedlings and was represented as stomatal index (SI; the ratio of the number of stomata to total epidermal cells in an area). As shown in Fig. 1 (A to D and I), single *snrk2.2* and *2.3* mutants, but not *snrk2.6*, displayed modest but statistically significant increase in SI. All three of the double mutants, particularly *snrk2.2/2.3*, exhibited noticeably higher SIs than the single mutants, and the triple *snrk2.2/2.3/2.6* mutants showed the highest and most marked increase in SI (Fig. 1, E to H and I). However, we did not observe notable stomatal clustering in the triple mutants, suggesting that the mechanisms that guard against patterning defects were still effective. Further, the rescue of the triple *snrk2* mutants by a genomic fragment of *SnRK2.6* supports that the stomatal phenotype is dependent on *SnRK2*s (Fig. 1J and fig. S1A). The size of the cotyledons examined at this relatively young stage was also not significantly different compared to WT (fig. S1C). In addition, we generated a transgenic *Arabidopsis* line that overexpresses *SnRK2.6*, and it produced substantially fewer stomata and had lower SI (Fig. 1N and fig. S1B). Thus, our mutant analyses show that the three *SnRK2*s suppress stomatal formation redundantly.

Next, we asked whether ABA regulates stomatal development through these *SnRK2*s. WT and the triple *snrk2* mutant seedlings [3 days post-germination (dpg)] were transferred to and grown on medium containing increasing concentrations of ABA (i.e., 0, 0.05, 0.1, 0.3, 0.6, and 1.0 μM) for seven more days. Like previous reports, WT seedlings displayed stunted growth and yellowing leaves at high ABA concentrations, but the triple *snrk2* mutants were resistant to the ABA effect (fig. S1D) (*9*, *10*). We then examined in detail the SI of WT and *snrk2.2/2.3/2.6* exposed to these ABA concentrations. In WT, stomatal production was negatively correlated with ABA, i.e., plants displayed lower SIs when grown at higher ABA concentrations (Fig. 1, K to M and R). In contrast, the elevated level of stomatal production in *snrk2.2/2.3/2.6* was not affected by ABA and the mutant maintained its SI over the tested range of ABA concentration (Fig. 1, O to R). These results demonstrate that the negative effect of ABA on stomatal development is mediated through the three *SnRK2*s.

### Early stomatal lineage–expressed *SnRK2*s suppress stomatal production

**Fig. 2.**
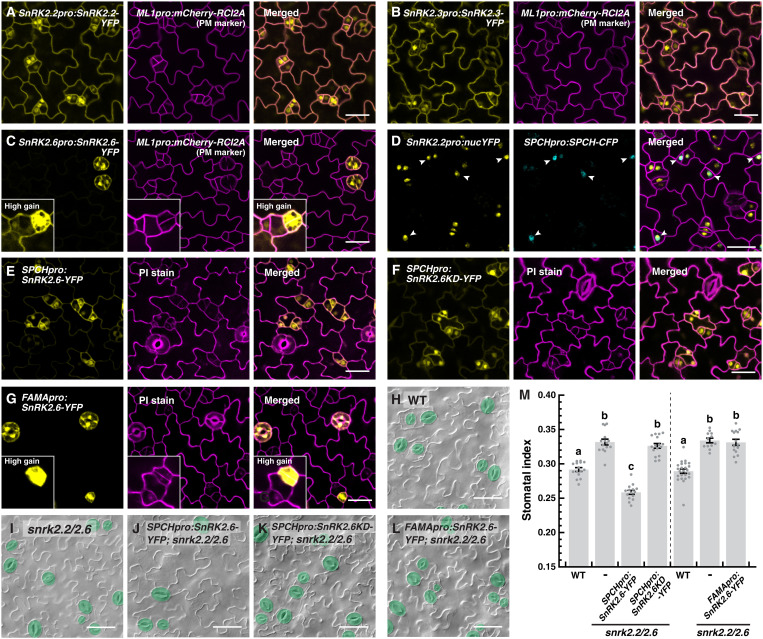
Stomatal lineage–expressed *SnRK2.2/2.3/2.6* influence stomatal production at the early, but not late, stage of stomatal development. (**A** to **C**) Expression pattern of the translational reporters of *SnRK2.2/2.3/2.6*. Confocal images of 3-day-old abaxial cotyledons of *SnRK2.2pro:SnRK2.2-YFP* (A), *SnRK2.3pro:SnRK2.3-YFP* (B), and *SnRK2.6pro:SnRK2.6-YFP* (C) (yellow). (**D**) Coexpression of *SnRK2.2* and *SPCH*. Confocal analysis of 3-day-old abaxial cotyledons of a transgenic seedling harboring both *SnRK2.2pro:nucYFP* (yellow) and *SPCHpro:SPCH-CFP* (cyan). Arrowheads marked the early stomatal lineage cells with noticeable coexpression of both genes. (**E** to **G**) Stomatal lineage stage–specific expression of *SnRK2.6* and its variant driven by the promoter of *SPCH* and *FAMA*. Confocal analysis of 3-day-old abaxial cotyledons of *SPCHpro:SnRK2.6-YFP* (E), *SPCHpro:SnRK2.6KD-YFP*, which encodes a kinase-dead version of SnRK2.6 (F) (see Materials and Methods), and *FAMApro:SnRK2.6-YFP* (G). (**H** to **M**) Genetic complementation test of the stage-specific *SnRK2.6* lines. Representative images (H to L) and quantification of stomatal indices (M) of 10-day-old abaxial cotyledons of wild-type (WT; H), *snrk2.2/2.6* (I), and *snrk2.2/2.6* transformed with *SPCHpro:SnRK2.6-YFP* (J), *SPCHpro:SnRK2.6KD-YFP* (K), and *FAMApro:SnRK2.6-YFP* (L). Stomata are pseudo-colored in green. Values are means ± SEM; *n* ≥ 12 independent cotyledons. One-way ANOVA with Tukey’s multiple comparisons test, *P* < 0.05. Cell outlines [plasma membrane (PM)] are marked by *ML1pro:mCherry-RCI2A* (magenta; A to D) or propidium iodide (PI) staining (E to G). Scale bars, 25 μm (A to G) or 50 μm (H to L).

To understand how the *SnRK2*s influence stomatal development, we studied their potential expression in the stomatal lineage and their subcellular localization. We generated translational reporter lines of the three *SnRK2*s [yellow fluorescent protein (YFP)–tagged SnRK2s driven by their native promoters], as well as transcriptional reporters for *SnRK2.2* and *SnRK2.6*, and examined their expression patterns by confocal microscopy. We found that two of the *SnRK2*s, *2.2* and *2.3*, were broadly expressed on the epidermis of the cotyledons, including the stomatal precursor cells (Fig. 2, A and B). On the other hand, *SnRK2.6* had a more restrictive expression pattern with highest levels in mature guard cells, similar to the published results from a *GUS* reporter line (Fig. 2C and fig. S2A) (*4*). Nevertheless, at high exposure, we detected low levels of *SnRK2.6* expression in early stomatal lineage and epidermal cells (Fig. 2C, insets). The expression in these cells was notably absent when *SnRK2.6* was specifically driven to be expressed at the late stage of stomatal development by using the *FAMA* promoter (Fig. 2G, insets). Stomatal development involves a series of cell stage transitions, and *FAMA* is expressed during the last transition from guard mother cells to guard cells (*31*). Further, at an earlier stage during seedling growth (2 dpg), we observed broad expression of *SnRK2.6* in all epidermal cells of the cotyledons, suggesting that the expression of *SnRK2.6* becomes more restrictive as the organ matures (fig. S2B). As for their subcellular localization, the SnRK2s are present in the nucleus and appear to associate with the plasma membrane (Fig. 2, A to C). However, close examinations and comparison with the tonoplast marker suggest that the membrane-like localization could be cytoplasmic species of the proteins being “pushed” to the plasma membrane by the vacuoles in the cells (fig. S2C). To further confirm the expression of *SnRK2*s in the stomatal precursors, we generated a dual reporter line that contains both a *SPCH* translational reporter (*SPCHpro:SPCH-CFP*), which marks the stomatal precursors (*19*), and *SnRK2.2pro:nucYFP*. Through confocal analyses, we found that all *SPCH*-expressing cells also express *SnRK2.2* (Fig. 2D, arrowheads). These results indicate that the *SnRK2*s are present in the stomatal precursors and the kinases may directly regulate these cells.

To test whether the stomatal precursor-expressed SnRK2 proteins are sufficient to affect stomatal development, we generated reporter constructs of *SnRK2*.*6* under either *SPCH* or *FAMA* promoter to drive *SnRK2*.*6* expression in the early or late stage of stomatal development, respectively. A kinase-dead version of *SnRK2.6*, *SnRK2.6KD* (see Materials and Methods) (*32*), under *SPCHpro*, was also constructed to test whether its kinase activity is required. The constructs were then transformed into the double mutant *snrk2.2/2.6* to test for complementation. We first examined the expression pattern of the lines and confirmed the enrichment of the YFP-tagged SnRK2.6 and SnRK2.6KD in the targeted cell stages (Fig. 2, E to G). Phenotypic analyses of these lines showed that *SPCHpro:SnRK2.6-YFP* not only suppressed the higher SI of *snrk2.2/2.6* but also led to further reduction in its SI, compared to WT (Fig. 2, H to J and M). This highlights the functional consequence of having enhanced levels of SnRK2.6 in early stomatal precursor cells. However, neither *SPCHpro:SnRK2.6KD-YFP* nor *FAMApro:SnRK2.6-YFP* were able to rescue the stomatal defect (Fig. 2, K to M). Thus, the cell stage–specific complementation analyses suggest that the SnRK2s expressed in the early, but not late, stomatal lineage cells regulate stomatal development, and the influence is dependent on the kinase activity.

### SnRK2.2/2.3/2.6 directly interact and phosphorylate SPCH at distinct residues

**Fig. 3.**
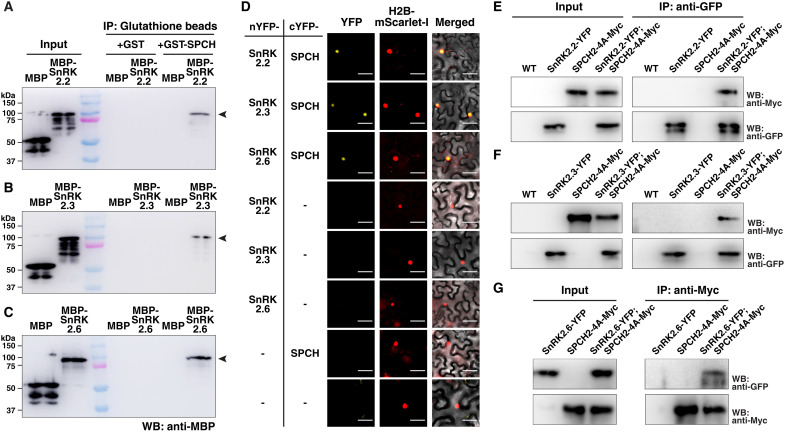
SnRK2.2/2.3/2.6 interact with SPCH in vitro and in vivo. (**A** to **C**) In vitro GST pull-down assays between recombinant MBP-tagged SnRK2s and GST-SPCH. MBP and MBP-fused SnRK2.2 (A), 2.3 (B), and 2.6 (C) were incubated with immobilized GST or GST-SPCH. Immunoprecipitated (IP) fractions were probed with an anti-MBP antibody. (**D**) Bimolecular fluorescence complementation (BiFC) analyses between SnRK2s and SPCH. N- and C-terminal fragments of yellow fluorescent protein (nYFP and cYFP) were fused to SnRK2.2/2.3/2.6 and SPCH, respectively. The presence of YFP signal (yellow) indicates protein interaction between the test pairs. Positions of the nuclei (red) were marked by H2B-mScarlet-I, stably expressed in *N. benthamiana*. (**E** to **G**) In vivo coimmunoprecipitation assays between SnRK2s and SPCH. Transgenic *Arabidopsis* expressing either the native promoter-driven, *YFP*-fused *SnRK2*, i.e., *2.2* (E), *2.3* (F), and *2.6* (G), *SPCHpro:SPCH2-4A-MYC,* or both, were grown for 3 days (E and F) or 2 days (G), and total soluble proteins were extracted for immunoprecipitation by anti-GFP (E and F) or anti-Myc (G) beads. Fractions were probed by anti-Myc and anti-GFP antibodies. Scale bar, 50 μm.

The above analyses hinted that the SnRK2s can target specific protein regulator(s) in the stomatal precursors to affect stomatal production. Since SnRK2s are enriched in the nucleus of stomatal precursors, we speculated that they may directly interact and phosphorylate the nuclear-localized SPCH. To test whether the three SnRK2s and SPCH interact, recombinant maltose-binding protein (MBP)–tagged SnRK2.2, 2.3, and 2.6 and glutathione *S*-transferase (GST)–tagged SPCH were produced in bacteria and were used in in vitro pull-down assays. We found that GST-SPCH, but not GST alone, can pull down MBP-SnRK2.2, 2.3, and 2.6, and GST-SPCH does not interact with MBP (Fig. 3, A to C). Next, using the transient expression system in *Nicotiana benthamiana*, we performed bimolecular fluorescent complementation (BiFC) assays between the SnRK2s and SPCH. While the control protein pairs did not produce any signal, coexpression of nYFP-SnRK2.2, 2.3, or 2.6 with cYFP-SPCH resulted in nuclear-localized YFP signals, indicating that the SnRK2s and SPCH interact in the nucleus (Fig. 3D). Further, we tested in vivo interaction between them by coimmunoprecipitation. The translational YFP reporter lines of the three *SnRK2*s in *Arabidopsis* described earlier were crossed with *SPCHpro:SPCH2-4A-Myc*, which encodes a stabilized form of SPCH driven by its own promoter (see below) (*20*, *26*). The use of this version of SPCH was needed because the expression level of cell type–specific SPCH is too low for the biochemical-based assay. As shown in Fig. 3 (E and F), YFP-tagged SnRK2.2 and 2.3 successfully pulled down the Myc-tagged SPCH only in the double tagged lines, but not in the control lines. For the assay between SnRK2.6 and SPCH, 2-dpg seedlings, where *SnRK2.6* is expressed in early stomatal precursors (fig. S2B), were used, and we also observed interaction between the proteins (Fig. 3G). Thus, these in vitro and in vivo interaction assays strongly suggest that the three SnRK2s can directly interact with SPCH.

**Fig. 4.**
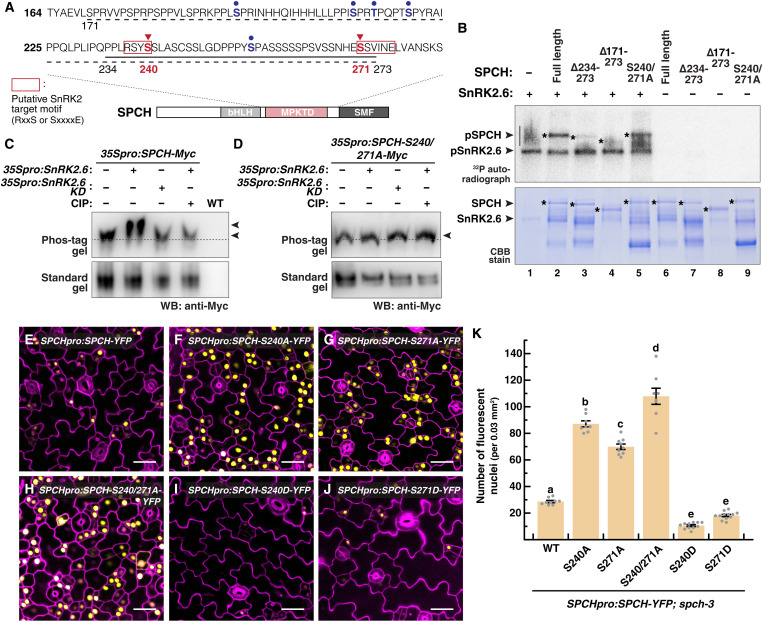
SnRK2.2/2.3/2.6 directly phosphorylate SPCH at distinct, stability-altering residues. (**A**) Putative SnRK2 phosphorylation sites on SPCH. SPCH contains a MAPK target domain (MPKTD) where five residues (blue circles) are targets of MPK3 and MPK6. Two putative SnRK2 phosphorylation residues (S240 and S271; red triangles) were highlighted on the basis of predicted SnRK2 motifs (bracket). Deleted regions [in (B)] are denoted as a short solid underline (∆234–273) and a long dashed underline (∆171–273). (**B**) In vitro phosphorylation assay with ^32^P-labeled ATP. Recombinant SPCH and its variants were incubated alone or with recombinant SnRK2.6. Upper panel shows the autoradiograph, and the bottom was Coomassie blue (CBB) protein staining. |: Probable phosphorylated species (nonspecific) in the absence of substrate. *: Full-length or truncated/mutated forms of SPCH proteins. (**C** and **D**) In vivo phosphorylation assays in *N. benthamiana*. *35Spro:SPCH-MYC* (C) and *35SproSPCH-S240/271A-MYC* (D) were transiently expressed in leaves together with either *35Spro:SnRK2.6* or *SnRK2.6KD* (its kinase-dead version; see Materials and Methods). Protein extracts, with or without treating with alkaline phosphatase (CIP), were analyzed on a Phos-tag (upper) or a standard (lower) SDS-PAGE gel and probed with an anti-Myc antibody. (**E** to **K**) Analysis of translational reporters of *SPCH* and its phospho-variants. Confocal images of 3-day-old abaxial cotyledons of *SPCHpro:SPCH-YFP* (E), its phospho-dead variants S240A (F), S271A (G), and S240/271A (H), and its phospho-mimetic variants S240D (I) and S271D (J) in *spch-3*. Images were taken with the same excitation and acquisition settings. The numbers of YFP-expressing cells (yellow) are quantified (K). Values are means ± SEM; *n* ≥ 6 independent cotyledons. One-way ANOVA with Tukey’s multiple comparisons test, *P* < 0.05. Cell outlines were visualized with propidium iodide (magenta). Scale bar, 25 μm.

Phosphoproteomics studies have revealed putative motifs that are targeted by SnRK2 kinases (*33*, *34*). Intriguingly, we identified one each of the two putative SnRK2-targeted motifs, RxxS and SxxxxE, as defined by Umezawa *et al.* (*33*), within the mitogen-activated protein kinase (MAPK) target domain (MPKTD) of SPCH (Fig. 4A) (*26*). This domain was originally defined as an SPCH-specific region where multiple residues within are targeted by MPK3 and MPK6 (Fig. 4A, blue circles). Notably, although the two putative SnRK2 phosphorylation sites (S240 and S271) are within the same MPKTD region, they do not overlap with the known target sites of the MAPKs.

We first tested whether the SnRK2s can phosphorylate SPCH by radioactive in vitro kinase assays. SnRK2.6 was used as the representative SnRK2, and recombinant SnRK2.6, alone or with either full-length or truncated/mutated versions of SPCH, was incubated with ^32^P-labeled adenosine 5′-triphosphate (ATP). Our SnRK2.6 was active and can phosphorylate both itself and full-length SPCH (Fig. 4B, lanes 1 and 2, upper panel, and fig. S3 for replicate). The smear of higher–molecular weight species in the kinase alone control (vertical line, lane 1), which is absent in lane 2, may represent nonspecific phosphorylation of proteins in the reaction mix by SnRK2.6 when its preferred substrate was not present. In addition, the kinase was not effective in phosphorylating the shorter (∆234–273) and full (∆171–273) MPKTD truncated versions of SPCH, suggesting that its 234– to 273–amino acid region, which contains the two putative SnRK2 target sites, is critical for SPCH phosphorylation by SnRK2.6 (lanes 3 and 4). However, we found that SPCH-S240/271A, where both the SnRK2 target sites were substituted to alanine, can still be phosphorylated by SnRK2.6 (lane 5). This is not entirely surprising, since there are a few neighboring serine residues that may act as secondary phosphorylation sites. Using mass spectrometry, we found evidence of phosphorylation at S240 using WT SPCH, but with SPCH-S240/271A, we detected phosphorylation at S241 instead (fig. S4). Further, the high background signals in the SPCH-S240/271A sample suggest that SPCH-S240/271A is a less preferred substrate than WT SPCH (Fig. 4B and fig. S3, lane 5 versus lane 2).

In addition, we examined whether SnRK2 can direct SPCH phosphorylation in plant cells. Myc-tagged SPCH was coexpressed with SnRK2.6 or its kinase-dead version SnRK2.6KD (*32*) transiently in *N. benthamiana*, and the protein extracts were treated with either calf intestinal alkaline phosphatase (CIP) or mock. Phos-tag immunoblot analyses showed that the coexpression of SnRK2.6 led to a shift in the mobility of SPCH, which was absent in the SnRK2.6KD coexpressed sample or after CIP treatment (Fig. 4C). This shows that SPCH can be phosphorylated by SnRK2.6 in planta. Notably, the mobility shift was not observed for SPCH-S240/271A, indicating the importance of the two predicted sites for SnRK2 phosphorylation (Fig. 4D). The higher stringency of the kinase in planta, as compared to the in vitro kinase assay, may be due to the differences in protein concentration and/or the presence of other protein factor(s) in vivo that led to increased specificity.

To assess the functional importance of the two SnRK2 sites on SPCH protein in vivo, the phospho-dead (S240A, S271A, and S240/271A) and phospho-mimetic (S240D and S271D) versions of SPCH were used to construct YFP translational reporters (under *SPCH* promoter and in *spch-3*), and their phenotypes were examined by confocal microscopy. Compared with the WT *SPCH* reporter, the expression of the phospho-dead versions, particularly S240/271A, was substantially higher, and they drove overproliferation of meristemoid-like cells and produced higher number of YFP-positive cells (Fig. 4, E to H and K, and fig. S5A). In line with the *snrk2* triple mutants and the overexpression of WT SPCH (*19*, *35*), however, no severe stomatal clustering was observed. In contrast to the phospho-dead variants, the phospho-mimetic versions were expressed in fewer cells and the signal intensity of the S240D variant was markedly lower (Fig. 4, I to K, and fig. S5A). Further, although all the variants rescued the seedling lethal phenotype of *spch-3*, this S240D variant produced far fewer stomata than WT, which is in line with its reduced stability (fig. S6). The results suggest that the two SnRK2-targeted sites are functionally relevant in vivo and can dictate SPCH stability and activity.

### SnRK2-mediated phosphocode transduces ABA signals in regulating SPCH, stomatal development, and drought tolerance

**Fig. 5.**
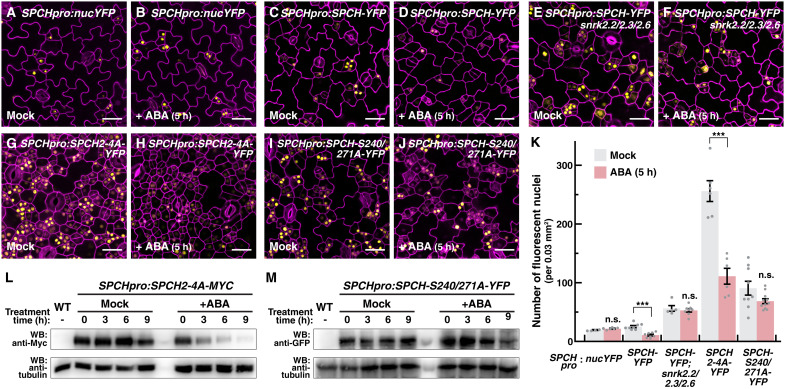
SnRK2-targeted residues on SPCH transduce ABA-mediated suppression of SPCH. (**A** to **K**) Effect of ABA on the transcriptional and translational reporters of *SPCH* and its phospho-variants. Confocal images of 3-day-old abaxial cotyledons of *SPCHpro:nucYFP* (A and B), *SPCHpro:SPCH-YFP* (C and D), *SPCHpro:SPCH-YFP* in *snrk2* triple mutant (E and F), *SPCHpro:SPCH2-4A-YFP* (G and H), and *SPCHpro:SPCH-S240/271A-YFP* (I and J) treated with mock (A, C, E, G, and I) or ABA (60 μM; B, D, F, H, and J) for 5 hours. Images were taken with the same excitation and acquisition settings. The numbers of YFP-expressing cells (yellow) are quantified (K). Values are means ± SEM; *n ≥* 5 independent cotyledons. One-way ANOVA with Tukey’s multiple comparisons test, *P* < 0.05. Cell outlines were visualized with propidium iodide (magenta). Scale bar, 25 μm. (**L** and **M**) Changes in the protein levels of SPCH phospho-variants in response to ABA. Three-day-old seedlings of *SPCHpro:SPCH2-4A-MYC* (L) and *SPCHpro:SPCH-S240/271A-YFP* (M) were transferred to medium without (Mock) or with ABA (60 μM), and samples were harvested at the indicated time for Western analyses with the indicated antibodies.

We next examined how ABA regulates *SPCH* expression and whether the SPCH phospho-variants have altered response to ABA. Transcriptional and translational reporters of *SPCH* and its variants were subjected to short-term ABA treatment, and their expression was assayed by confocal microscopy. Our ABA treatment did not generate a notable response in the transcriptional reporter *SPCHpro:nucYFP* in the assayed time frame, but the expression level of SPCH proteins and the number of YFP-positive cells were significantly reduced in *SPCHpro:SPCH-YFP*, suggesting that ABA suppresses *SPCH* expression at the protein level (Fig. 5, A to D and K, and fig. S5B). In line with this, we found that the addition of MG132, a proteasome inhibitor, blocked the effect of ABA on SPCH in *SPCHpro:SPCH-YFP*, and the addition of ABA also led to down-regulation of SPCH proteins when the constitutive 35*S* promoter was used to drive *SPCH* in a time-series immunoblot analysis (figs. S7 and S8). The suppression of SPCH by ABA is also dependent on the three SnRK2s, as *SPCHpro:SPCH-YFP* in the triple *snrk2* background did not respond to ABA (Fig. 5, E, F, and K).

We further tested the ABA response of the SnRK2-resistant SPCH-S240/271A variant and, for comparison, the SPCH2-4A variant. The latter variant contains alanine substitutions at three phosphorylation sites (S211, T214, and S219) targeted by MPK3 and MPK6, rendering it resistant to MAPK signaling, and this variant, like SPCH-S240/271A, drives overproliferation of stomatal precursors (Fig. 5G) (*26*). Despite being a stabilized, overactive variant, the levels of SPCH2-4A were effectively down-regulated by ABA (Fig. 5, G, H, and K, and fig. S5B). Strikingly, in contrast, SPCH-S240/271A displayed resistance toward ABA, having modest reduction in YFP-expressing cells upon ABA treatment (Fig. 5, I to K). These observations were also supported by our time-course immunoblot analyses, where, in response to ABA, the level of SPCH2-4A protein declined in the first few hours, but the level of SPCH-S240/271A remained high and only started to fall at later time points (Fig. 5, L and M).

**Fig. 6.**
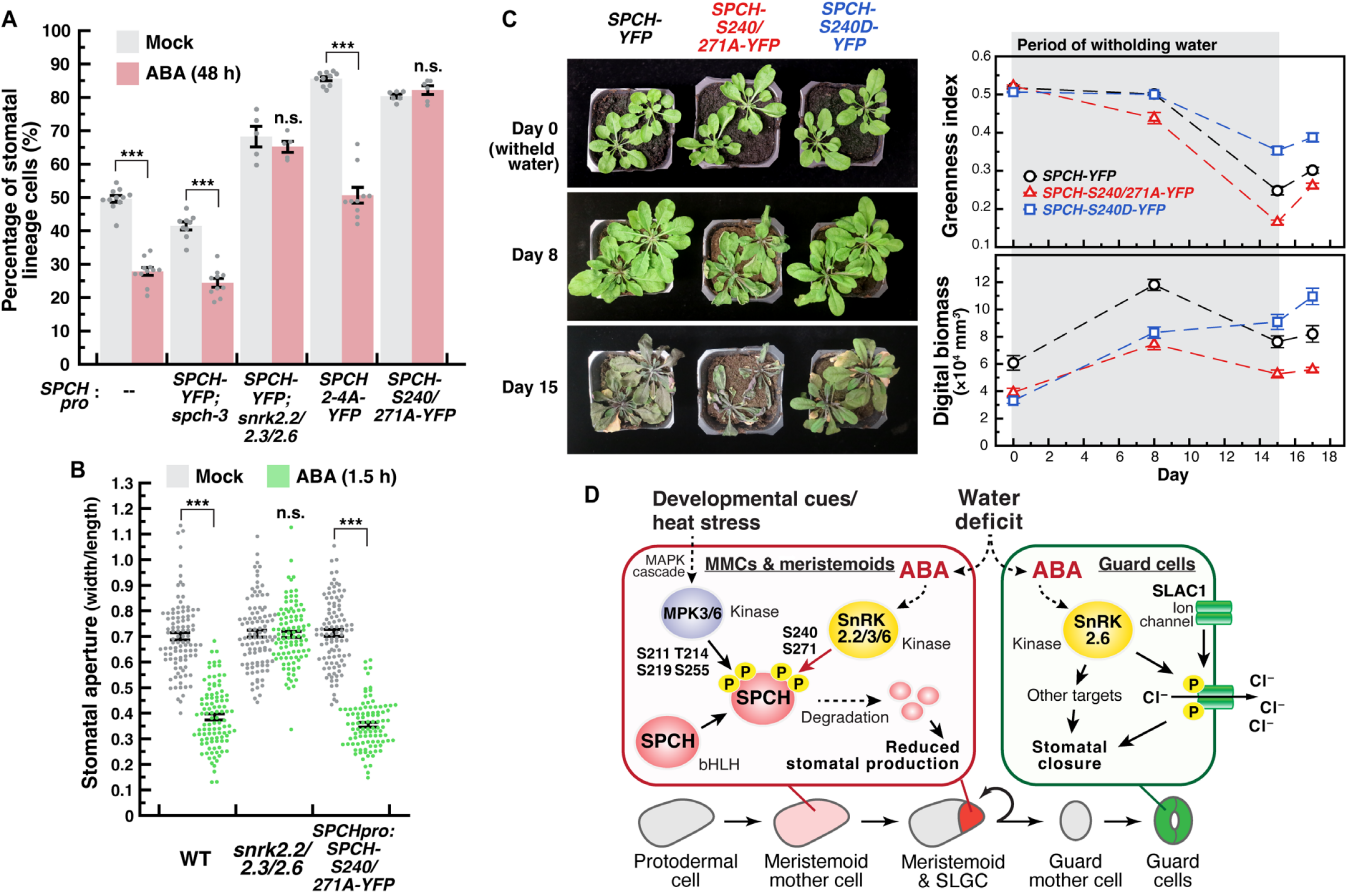
SnRK2-mediated phosphocode on SPCH regulates stomatal development in response to ABA and alters drought tolerance in adult plants. (**A**) Effect of ABA on the stomatal lineage cell population of SPCH reporters and its phospho-variants. Seedlings of WT, *SPCHpro:SPCH-YFP* in *spch-3*, *SPCHpro:SPCH-YFP* in *snrk2* triple mutant, *SPCHpro:SPCH2-4A-YFP*, and *SPCHpro:SPCH-S240/271A-YFP* were grown for 2 days before transferring to medium without (Mock) or with ABA (1 μM) for two more days. The epidermal cells of the abaxial cotyledons were imaged and quantified. (**B**) Effect of ABA on stomatal aperture of WT, *snrk2.2/2.3/2.6*, and *SPCHpro:SPCH-S240/271A-YFP*. Seedlings (5 dpg) were incubated with stomatal opening buffer before treatment with mock or ABA (10 μM) for 1.5 hours. Values are means ± SEM; *n* ≥ 10 independent cotyledons (A) or 100 stomata (B). Student’s *t* test, *P* < 0.01. (**C**) Drought tolerance assay. Four-week-old *SPCHpro:SPCH-YFP*, *SPCHpro:SPCH-S240/271A-YFP* (phospho-dead), and *SPCHpro:SPCH-S240D-YFP* (phospho-mimetic) (all in *spch-3* background) were subjected to drought stress for 15 days (water was withheld at day 0) before rewatering at day 15. The greenness index and digital biomass were captured by the 3D multispectral scanner PlantEye (Phenospex) on the indicated days. Values are means ± SEM; *n* = 10 pots of two plants (imaged as a unit). (**D**) Model of the effect of ABA on stomatal development. ABA, whose level increases in response to drought, activates the SnRK2 kinases in the early stomatal precursor cells. The activated kinases then phosphorylate SPCH at specific residues, leading to their degradation and reduced stomatal production. In mature guard cells, activated SnRK2.6/OST phosphorylates ion channels, such as SLAC1, and closes the stomatal pore.

We also checked how long-term ABA treatment can affect stomatal development among the *SPCH* reporter lines. Since both the SPCH phospho-dead variants overproduce stomatal precursors, accurate phenotyping of their SI at later stage is not feasible. Thus, we counted stomatal lineage cells on the cotyledons of 4-dpg seedlings (see Materials and Methods), which were treated with ABA or mock for 48 hours prior, by confocal microscopy. As expected, WT and *SPCHpro:SPCH-YFP* produced fewer stomatal lineage cells in the presence of ABA, and the response is dependent on the SnRK2 kinases (Fig. 6A). Consistent with the short-term treatment results above, we further found that ABA suppressed stomatal development in SPCH2-4A, but SPCH-S240/271A was resistant to the inhibition. Also, this insensitivity of SPCH-S240/271A toward ABA occurs only at the level of stomatal development, as its stomatal closing response was not affected (Fig. 6B). These results demonstrate that ABA-mediated suppression of SPCH occurs at the protein level and is related through the phosphorylation of the two serine residues on SPCH by the SnRK2 kinases.

Last, we tested how the SnRK2-targeted residues may influence SPCH levels and overall fitness of adult plants during water shortage. Four-week-old *Arabidopsis* of the phospho-dead and phospho-mimetic SPCH variants, i.e., SPCH-S240/271A and SPCH-S240D, respectively, together with WT SPCH, were subjected to drought treatment. At day 6 after withholding water, we found a significant decrease in *SPCH* expression in the emerging leaves of the WT SPCH line, while the level of SPCH-S240/271A and SPCH-S240D remained high and low, respectively (fig. S9, A to G). The results also correlate with their stomatal density at a later stage (fig. S9H). To monitor plant vigor, we documented plants at the indicated intervals from the start of the treatment, both visually and using a multispectral three-dimensional (3D) scanner (Phenospex PlantEye, which captured their greenness index and digital biomass; see Materials and Methods) (Fig. 6C). Compared to the WT SPCH line, the phospho-dead SPCH-S240/271A variant (red) was hypersensitive to drought but the phospho-mimetic SPCH-S240D (blue) was more tolerant. These results demonstrate that the SnRK2-mediated SPCH phosphocode is functionally relevant in mediating the stomatal response to drought and can modulate drought tolerance.

## DISCUSSION

Overall, our genetic and biochemical analyses reveal a direct link between the core ABA signaling kinases and the master stomatal regulator SPCH that exists within the early stomatal precursors, providing mechanistic insights into a key response for water conservation in plants (Fig. 6D). When ABA levels are high, e.g., because of water shortage, the SnRK2s present in the stomatal precursors are activated and directly phosphorylate SPCH at S240 and S271. The phosphorylated SPCH is then suppressed, likely through protein degradation, which leads to inhibition of stomatal development. When water supply resumes, ABA levels drop and SnRK2s are inactivated, relieving their inhibition on SPCH and stomatal production. This connection allows ABA signaling to directly influence the activity of the stomatal precursors and hence stomatal number.

Although an early report in *Arabidopsis* suggested that exogenous application of ABA promotes stomatal production, our work, together with two other studies that analyzed ABA metabolic and perception mutants, confirms the negative role of ABA on stomatal development (*6*, *29*, *36*). By examining higher-order mutants of the *SnRK2* family, our work also clarifies the importance of the subclass III SnRK2s, including SnRK2.6 (*30*), in the ABA-mediated suppression of stomatal production. From the perspective of the overall stomatal responses regulated by ABA, plants, using only the core SnRK2 kinases, can translate the generic ABA signals into the two distinct stomatal responses by targeting critical cell type–specific effectors, i.e., SPCH in meristemoids and SLAC1 and other substrates in guard cells, to coordinate the water conservation responses (Fig. 6D).

Protein phosphocodes refer to a set of phosphorylated residues of a protein that is “encoded” by a specific kinase(s) and/or “decoded” by specific signaling molecules (*37*–*40*). Both MAPKs and BIN2, a kinase that mediate brassinosteroid signaling and stomatal asymmetric cell division, suppress SPCH proteins, and they appear to “write” a similar code on SPCH, at least within its MPKTD region (*22*, *26*, *41*). The phosphorylation of SPCH by SnRK2s, however, occurs at two novel and previously uncharacterized residues at the same MPKTD region (Fig. 4A). The fact that the two residues, S240 and S271, but not the other MAPK-targeted sites, are necessary to transduce the inhibitory ABA signals suggests that they represent an ABA-specific phosphocode and that distinct signaling pathways could generate signal-specific codes on SPCH. Besides the two destabilizing kinases, the phosphorylation of SPCH at other residues by CDKA;1 and KIN10 promotes SPCH stability (*27*, *42*). How the different SPCH phosphocodes are decoded into a similar or different outcome and whether phosphorylation by one type of the kinases affects the “encoding” by other kinases are some exciting questions that remain to be addressed.

Water deficit, besides inducing ABA signaling, can trigger ABA-independent osmotic stress response, and the subclass III SnRK2s, together with other family members, are also key regulators of this process (*43*–*45*). Recent identification of the early osmotic stress-triggered Raf-like kinases that directly activate SnRK2s further underscores the importance of SnRK2s in the osmotic stress pathway (*46*–*48*). In line with this, when we subjected our *SPCH* reporter lines to 200 mM mannitol, we found that the SnRK2-insensitve SPCH-S240/271A variant was resistant to the osmotic stress treatment, while WT SPCH and SPCH2-4A remained sensitive (fig. S10). The results support that the SnRK2-mediated phosphocode also transduces osmotic stress signals in suppressing SPCH. It is worth noting, however, that a previous study proposed that the MAPK-dependent regulation of SPCH is involved in the osmotic response (*49*). The discrepancy might be explained by the extent of the variant’s sensitivity—SPCH2-4A was, compared to WT SPCH, more visible after the treatment, likely owing to its higher expression level (fig. S10I). In addition, the SPCH1-4A variant used in the previous study may have an altered response beyond being insensitive to MAPK signaling. In contrast to the suppressive role of MAPKs, the S193 residue, i.e., the “1” site in SPCH1-4A, has a distinct positive role in promoting stomatal development and may be a target of other unknown pathway(s) (*26*).

In addition to drought, osmotic stress can be caused by high salinity and low temperature, and it will be interesting to study how these stresses affect stomatal development and if the SnRK2-dependent SPCH regulation plays a role. Under cold stress, SnRK2.6/OST1 is activated and it promotes cold tolerance by phosphorylating and stabilizing the bHLH transcription factor ICE1/SCRM, a positive regulator of this response (*50*, *51*). This is intriguing because ICE1 also functions as the heterodimeric partner of SPCH (*52*). However, since SnRK2.6 stabilizes ICE1 in freezing tolerance, this positive relationship would rule out its contribution to the SnRK2-mediated suppression of stomatal development, where SnRK2.6 and ICE1 have opposing roles. Unlike *SPCH*, *ICE1* is more broadly expressed and acts as homodimers in promoting freezing tolerance (*51*). The distinct cellular and molecular context of ICE1 in these processes could result in independent modes of regulation.

Through the drought stress experiment, we demonstrate the significance of the SnRK2-specific SPCH phosphocode in the response of SPCH and stomatal formation toward water deficit and its impact on drought tolerance (Fig. 6C and fig. S9). By manipulating this phosphocode, the water-dependent plasticity of stomatal production may be fine-tuned, with the potential benefit of retaining plasticity toward other endogenous/external signals, such as heat stress. Like the MPKTD region itself, the S240 and S271 residues are present in many dicots species (fig. S11). Our findings also show that the modulation of stomatal production by ABA can be uncoupled from ABA-induced stomatal closure through either the SPCH phosphocode or cell stage–specific complementation (Figs. 2M and 6, A and B, and fig. S12). The uncoupling of the two systems offers an opportunity to evaluate the contribution of each for drought tolerance and overall growth. From the perspective of crop improvement, having the ability to specifically control individual water-related stomatal responses may enable precise engineering of stomatal traits tailored to a variety of water conditions in traditional and urban farming.

## MATERIALS AND METHODS

### Plant materials and growth conditions

The *Arabidopsis thaliana* ecotype Columbia-0 (Col-0) was used as the WT, and all mutants and reporter lines in this study are in this background. Previously described mutants and reporter lines are as follows: *spch-3* (SAIL_36_B06) (*19*), *SPCHpro:SPCH2-4A-YFP* (*26*), *SPCHpro:SPCH2-4A-Myc* and *ML1pro:mCherry-RCI2A* (*20*), *snrk2.2* (*srk2d*) (GABI-Kat 807G04), *snrk2*.*3* (*srk2i*) (SALK_096546), *snrk2.6* (*srk2e*) (SALK_008068) (*9*, *53*), and a tonoplast reporter line, vac-ck (*54*).

*A. thaliana* seedlings were grown on ^1^/_2_ strength Murashige and Skoog agar medium at a light intensity of 80 μmol m^−2^ s^−1^ under long-day condition (16-hour light, 8-hour dark) at 22°C in a plant tissue culture chamber (Percival).

For long-term ABA treatment, seedlings (3 or 2 dpg, as indicated in the figures) grown on standard ^1^/_2_ Murashige and Skoog agar medium were transferred to ^1^/_2_ Murashige and Skoog agar medium containing ABA (Sigma-Aldrich, A1049) at the indicated concentrations and duration. For short-term ABA treatment, seedlings grown on standard ^1^/_2_ Murashige and Skoog agar medium were immersed with ^1^/_2_ Murashige and Skoog liquid medium containing the indicated concentration of ABA and were kept in the growth chamber for the said duration before microscopic analyses or protein extraction.

### Vector construction and plant transformation

To construct *SnRK2.2pro:SnRK2.2-YFP*, *SnRK2.3pro:SnRK2.3-YFP*, and *SnRK2.6pro:SnRK2.6-YFP*, full-length genomic regions including the 902-, 1496-, and 1620-bp promoters of the respective genes were cloned into pENTR/DTOPO vector and later introduced to plant binary vector *pHGY* (*55*) through LR recombination (LR Clonase II, Thermo Fisher Scientific; all the recombination procedures below were done using this method). Similarly, for *SnRK2.6pro:nucYFP*, the 1620-bp promoter of *SnRK2.6* was cloned into pENTR/DTOPO vector and recombined with the plant binary vector *pBGYN* (*55*). For *SPCHpro:SnRK2.6-YFP* and *SPCHpro:SnRK2.6KD-YFP*, gateway entry vectors that contain the 2.5-kbp promoter of *SPCH* (pDONR-P4-P1R) and the *SnRK2*.6 genomic region (start to stop codon, including introns) or its kinase site substituted version [K50N, introduced via mutagenesis polymerase chain reaction (PCR); table S1] (*32*) (pENTR/DTOPO) were recombined into the plant binary vector *R4pGWB540* (*56*). To construct *FAMApro:SnRK2.6-YFP*, the 2.5-kbp *FAMA* promoter was inserted upstream of the *SnRK2.6* genomic region (start to stop codon, including introns) in pENTR/DTOPO by NEBuilder HiFi DNA assembly (New England Biolabs) and recombined into the plant binary vector *pHGY* (*55*). To construct *SPCHpro:SPCH-YFP* and its phospho-variants, full-length genomic region including 2.5-kbp promoter of *SPCH* (without stop codon) was cloned into pENTR/DTOPO vector. This vector was then used to introduce the desired mutations at S240 and S271 on *SPCH* through site-directed mutagenesis PCR (table S1) and was later recombined with *pHGY* (*55*). To construct *35Spro:SPCH-MYC*, *SPCH* complementary DNA (cDNA) in pENTR/DTOPO was recombined with the binary vector *pGWB520* (*57*). The binary vectors were transformed into either Col-0, *ML1pro:mCherry-RCI2A*, or mutants as indicated through the floral-dip method (*58*). The *SPCH* promoter-driven SPCH and its variants were first transformed into *spch-3/+* (as homozygous *spch-3* is seedling lethal). Since *spch-3* is a SAIL (Syngenta Arabidopsis Insertion Library) T-DNA line containing a Basta-resistant gene, we used Basta resistance to follow and select for the *spch-3* allele. All the selected lines at T3 no longer segregate out the mutant allele and are homozygous for *spch-3*.

For constructing bacterial expression vectors for expressing MBP-SnRK2.2/2.3/2.6 and GST-SPCH, the cDNAs of the respective genes were cloned into pENTR/DTOPO and recombined into pETG-40A (for MBP-SnRK2s) or pDEST15 (for GST-SPCH), respectively. For the expression of TRX-SnRK2.6 and MBP-SPCH and its truncated/mutated variants for in vitro kinase assays, the coding sequence of *SnRK2.*6 and *SPCH* and its variants (i.e., ∆234–273, ∆171–273, and S240/271A) were cloned into the pET32a-TRXtag and pMAL-c2X expression vectors, respectively.

### Microscopy for fluorescent reporters, SI, and aperture analyses

Fluorescent reporter lines were analyzed by confocal microscopy. Fluorescent images were taken on an FV3000 confocal microscope (Olympus) and processed using ImageJ (National Institutes of Health). Cell outlines were visualized through the plasma membrane marker, *ML1pro:mCherry-RCI2A*, or propidium iodide (0.1 mg/ml; Thermo Fisher Scientific, P3566). Quantification of fluorescent nuclei was performed on ImageJ using the Cell Counter plug-in. For quantitative analyses of stomatal lineage cell populations in young seedlings (4 dpg), stomatal lineage cells, in addition to guard cells, were defined as having an area below 100 μm^2^, with their characteristic cell shape and without visible lobing.

For scoring SI, seedlings were fixed by immersing in fixing solution [7:1 (v/v) mix of EtOH:acetic acid] overnight and washed three times with 70% EtOH. Clearing solution [4:1:2 (w/v/v) mix of chloral hydrate:glycerol:water] was added, and samples were cleared overnight. Differential contrast interference (DIC) images were taken at ×20 on a Zeiss upright microscope (Axio Imager M2) equipped with a digital complementary metal-oxide semiconductor (CMOS) camera (Hamamatsu, Orca Flash4.0 v2). Quantification of cells was carried out using the Cell Counter plug-in in ImageJ.

For stomatal aperture measurements, whole seedlings (5 dpg) of the indicated genotypes were first submerged in stomatal opening buffer (50 mM KCl and 10 mM MES/KOH, pH 6.15) for 2 hours at 22°C under white light at 80 μmol m^−2^ s^−1^. ABA (final concentration: 10 μM; Sigma-Aldrich, A1049) or mock solution (methanol, solvent of ABA) was then added, and the seedlings were further incubated for 1.5 hours under the same conditions. Excised cotyledons were mounted on slides using the same incubating buffer, and light microscopic images of the abaxial epidermis were taken with the microscope system described above. The inner width and length of each stoma were measured by ImageJ, and stomatal apertures are represented as the ratio between the width and the length of a stoma.

### Recombinant protein expression and purification

ArcticExpress (DE3) cells (Agilent) were used as the expression host, and transformed cells were induced at low temperature (12°C, 180 rpm for 24 to 48 hours) with 0.5 mM isopropyl-β-d-thiogalactopyranoside. MBP-tagged proteins were purified with MBP amylose resin (New England Biolabs), GST-tagged proteins were purified with Pierce Glutathione Agarose (Thermo Fisher Scientific), and TRX-tagged proteins, which contain His-tag, were purified using Ni-NTA Agarose (Qiagen). For recombinant proteins used in the in vitro kinase assays, eluted proteins were loaded onto Sephadex G-25 resin HiPrep desalting columns and stored in a buffer containing 20 mM tris (pH 7.4), 50 mM NaCl, and 20% glycerol.

### In vitro pull-down assays

One microgram of bait protein GST-SPCH or 1 μg of GST control protein was first added to Pierce Glutathione Agarose (Thermo Fisher Scientific). One microgram of prey proteins, MBP-SnRK2.2/2.3/2.6, or MBP alone was then added to the GST-SPCH/GST–containing mix. Protein binding was conducted at 4°C on a rotator for 2 hours. Beads were washed five times with the wash buffer [20 mM tris-HCl (pH 7.5), 300 mM NaCl, 0.1% NP-40, and 1 mM EDTA]. Laemmli buffer (Bio-Rad) was added to the beads and boiled at 95°C for 15 min, and samples were analyzed by Western blot.

### BiFC assays

Full-length *SnRK2*s and *SPCH* were cloned into the respective plant binary vectors for BiFC, which encode either N- or C-terminal fragments of yellow fluorescent protein (nYFP or cYFP, respectively) upstream of the inserted gene and are driven by the 35*S* promoter. *Agrobacterium tumefaciens* (strain GV3101) carrying the indicated constructs were resuspended in the Infiltration buffer [10 mM MES (pH 5.7) and 10 mM MgCl_2_] and infiltrated into the lower surface of leaves from 4-week-old *N. benthamiana*. Leaf disks around the infiltrated regions were cut after 48 to 72 hours, and fluorescent signals were observed on a wide-field fluorescent microscope (Zeiss Axio Imager M2 equipped with a digital CMOS camera as described earlier).

### Coimmunoprecipitation assays

*Arabidopsis* seedlings of the indicated genotypes and stage were ground in liquid nitrogen, and total soluble proteins were extracted with immunoprecipitation buffer [50 mM tris-HCl (pH 7.5), 150 mM NaCl, 1 mM EDTA, 5% glycerol, 0.5% Triton X-100, 1 mM phenylmethylsulfonyl fluoride, proteinase inhibitor cocktail, and 25 μM MG132]. Green fluorescent protein (GFP)–Trap or Myc-Trap Magnetic Agarose (Chromotek) was used for immunoprecipitation. Bound proteins were eluted from the beads using Laemmli buffer (Bio-Rad) and analyzed by Western blot.

### Radioactive in vitro kinase assays

Approximately 500 ng of MBP-SPCH and its truncated/mutated variant proteins were incubated with or without 200 ng of TRX-SnRK2.6 in 20 μl of 20 mM tris-HCl (pH 7.4), 2.5 mM MgCl_2_, 2.5 mM MnCl_2_, 1 mM dithiothreitol (DTT), leupeptin (1 μg/ml), and 1 mM [γ^32^P]ATP. After 9 hours of incubation at room temperature, the kinase reactions were terminated by adding Laemmli sample buffer and boiling. Half of the samples were loaded to an SDS–polyacrylamide gel electrophoresis (SDS-PAGE) gel and stained with Coomassie Brilliant Blue. Incorporation of ^32^P into the proteins was detected from the SDS-PAGE gel using a phospho-screen.

### In planta phosphorylation assays

The indicated plant binary vectors were infiltered into the leaves of *N. benthamiana* as described in the BiFC assays above, and the infiltrated leaves were collected after 48 to 72 hours. Leaf samples were then ground to fine powders in liquid nitrogen, and total soluble proteins were extracted in an extraction buffer [50 mM tris-HCl (pH 7.5), 50 mM NaCl, 12 mM MgCl_2_, and 1 mM DTT]. CIP (New England Biolabs) was added to select extracts and incubated at 37°C for 1 hour. Reactions were terminated by adding Laemmli sample buffer and boiling. Protein samples were resolved on a standard or a Phos-tag (AAL-107, Wako Pure Chemical Corporation) 7.5% SDS-PAGE gel and analyzed by Western blot.

### Western blotting

Primary and secondary antibodies used in the immunoblotting are as follows: monoclonal anti-MBP antibody (New England Biolabs, E8032S, 1:10,000 dilution), monoclonal, horseradish peroxidase (HRP)–conjugated, anti-GST antibody (B-14) (Santa Cruz Biotechnology, sc-138 HRP, 1:1000), monoclonal anti-Myc antibody (Cell Signaling Technology, 71D10, 1:3000 dilution), monoclonal, HRP-conjugated, anti-GFP antibody (Miltenyi Biotec, 130-091-833, 1:3000 dilution), monoclonal anti–α-tubulin antibody (Santa Cruz Biotechnology, sc-5286 HRP, 1:1000), anti-mouse immunoglobulin G (IgG)–peroxidase antibody (Sigma-Aldrich, A9044, 1:5000), and anti-rabbit IgG, HRP-linked antibody (Cell Signaling Technology, 7074, 1:5000 dilution). Signal detection was performed with the SuperSignal West Dura Extended Duration Substrate (Thermo Fisher Scientific).

### Mass spectrometry

In vitro phosphorylation of SPCH and SPCHS240/271A proteins was conducted as described above, but with nonradioactive ATP. Protein samples were separated by SDS-PAGE on a 10% precast gel (Bio-Rad). Coomassie Brilliant Blue was used to locate the protein bands with the expected molecular weight. The gel bands were excised, cut into pieces of ~1 mm^3^, and transferred to the Sep-Pak tC18 96-well μElution Plate (Waters, Milford, MA, USA). The samples were then reduced and alkylated by 10 mM DTT and 55 mM iodoacetamide, respectively, with washing steps in between. After dehydration of the gel pieces, trypsin digestion was carried out at 37°C for 18 hours (1 μg of trypsin per 20 μg of protein), and the digested peptides were extracted with 70% acetonitrile and 0.1% trifluoroacetic acid (TFA).

Liquid chromatography–tandem mass spectrometry (LC-MS/MS) analysis was performed on the NanoLC-Ultra system with ChiPLC-nanoflex (Eksigent) in a trap-elute configuration. Desalted samples were loaded onto a 200 μm × 0.5 mm trap column and separated on a 75 μm × 150 mm analytical column (3 μm; ChromXP C18-CL, Eksigent). Peptide separation was done by a gradient solution of 2% acetonitrile, 0.1% formic acid (mobile phase A) and 98% ACN, 0.1% FA (mobile phase B) at a flow rate of 300 nl/min.

Peptide identification was carried out using the Mascot Server 2.6/7 software (Matrix Science). The acquired data were searched against the *A. thaliana* database on Swiss-Prot. Select search parameters are the following: type of search, MS/MS ion search; enzyme, trypsin; mass values, monoisotopic; protein mass, unrestricted; peptide mass tolerance, 100 ppm; maximum missed cleavages, 1; significance threshold, *P* < 0.004257; ion score or expect cutoff, 1. The LC-MS/MS and the data analyses were performed by the Protein and Proteomics Centre (PPC) at the National University of Singapore (NUS).

### Drought tolerance assay

Four-week-old *Arabidopsis* plants of the indicated genotypes were used in the assay. The drought treatment was conducted for 15 days, where day 0 marked the start of withholding water.

For microscopic analyses, the young, emerged leaves were marked at day 0. The true leaves that emerged thereafter were used for imaging at day 6 (confocal) and day 9 (DIC imaging).

For documenting plant fitness continually for the drought treatment, the Phenospex PlantEye, a multispectral 3D scanner, was used for the growth analysis at the indicated time points. For each genotype, 20 plants were analyzed (2 plants per pot, where a pot is scanned as a unit), and each scanning consisted of four successive scans where the pots were turned 90° each time. For the parameters for the greenness index and digital biomass, please refer to this website: https://phenospex.helpdocs.com/plant-parameters.

### Sequence alignment and statistical analyses of acquired data

The alignment of the SPCH protein sequences of the indicated species was generated by MUSCLE using MEGA7 with default parameters (*59*). The indicated statistical tests on our data were performed by Prism 9 (GraphPad).

## Notes

View/request a protocol for this paper from *Bio-protocol*.

## References

[1] A. M. Hetherington, F. I. Woodward, The role of stomata in sensing and driving environmental change. Nature 424, 901–908 (2003). 12931178 10.1038/nature01843

[2] S. R. Cutler, P. L. Rodriguez, R. R. Finkelstein, S. R. Abrams, Abscisic acid: Emergence of a core signaling network. Annu. Rev. Plant Biol. 61, 651–679 (2010). 20192755 10.1146/annurev-arplant-042809-112122

[3] T. H. Kim, M. Bohmer, H. Hu, N. Nishimura, J. I. Schroeder, Guard cell signal transduction network: Advances in understanding abscisic acid, CO_2_, and Ca^2+^ signaling. Annu. Rev. Plant Biol. 61, 561–591 (2010). 20192751 10.1146/annurev-arplant-042809-112226 PMC3056615

[4] A. C. Mustilli, S. Merlot, A. Vavasseur, F. Fenzi, J. Giraudat, Arabidopsis OST1 protein kinase mediates the regulation of stomatal aperture by abscisic acid and acts upstream of reactive oxygen species production. Plant Cell 14, 3089–3099 (2002). 12468729 10.1105/tpc.007906 PMC151204

[5] C. C. C. Chater, J. Oliver, S. Casson, J. E. Gray, Putting the brakes on: Abscisic acid as a central environmental regulator of stomatal development. New Phytol. 202, 376–391 (2014). 24611444 10.1111/nph.12713

[6] Y. Tanaka, T. Nose, Y. Jikumaru, Y. Kamiya, ABA inhibits entry into stomatal-lineage development in Arabidopsis leaves. Plant J. 74, 448–457 (2013). 23373882 10.1111/tpj.12136

[7] J. J. Weiner, F. C. Peterson, B. F. Volkman, S. R. Cutler, Structural and functional insights into core ABA signaling. Curr. Opin. Plant Biol. 13, 495–502 (2010). 20934900 10.1016/j.pbi.2010.09.007 PMC2971662

[8] T. Miyakawa, Y. Fujita, K. Yamaguchi-Shinozaki, M. Tanokura, Structure and function of abscisic acid receptors. Trends Plant Sci. 18, 259–266 (2013). 23265948 10.1016/j.tplants.2012.11.002

[9] Y. Fujita, K. Nakashima, T. Yoshida, T. Katagiri, S. Kidokoro, N. Kanamori, T. Umezawa, M. Fujita, K. Maruyama, K. Ishiyama, M. Kobayashi, S. Nakasone, K. Yamada, T. Ito, K. Shinozaki, K. Yamaguchi-Shinozaki, Three SnRK2 protein kinases are the main positive regulators of abscisic acid signaling in response to water stress in Arabidopsis. Plant Cell Physiol. 50, 2123–2132 (2009). 19880399 10.1093/pcp/pcp147

[10] H. Fujii, J. K. Zhu, Arabidopsis mutant deficient in 3 abscisic acid-activated protein kinases reveals critical roles in growth, reproduction, and stress. Proc. Natl. Acad. Sci. U.S.A. 106, 8380–8385 (2009). 19420218 10.1073/pnas.0903144106 PMC2688869

[11] H. Fujii, P. E. Verslues, J. K. Zhu, Identification of two protein kinases required for abscisic acid regulation of seed germination, root growth, and gene expression in *Arabidopsis*. Plant Cell 19, 485–494 (2007). 17307925 10.1105/tpc.106.048538 PMC1867333

[12] A. Joshi-Saha, C. Valon, J. Leung, A brand new START: Abscisic acid perception and transduction in the guard cell. Sci. Signal. 4, re4 (2011). 22126965 10.1126/scisignal.2002164

[13] D. Geiger, S. Scherzer, P. Mumm, A. Stange, I. Marten, H. Bauer, P. Ache, S. Matschi, A. Liese, K. A. S. al-Rasheid, T. Romeis, R. Hedrich, Activity of guard cell anion channel SLAC1 is controlled by drought-stress signaling kinase-phosphatase pair. Proc. Natl. Acad. Sci. U.S.A. 106, 21425–21430 (2009). 19955405 10.1073/pnas.0912021106 PMC2795561

[14] S. C. Lee, W. Lan, B. B. Buchanan, S. Luan, A protein kinase-phosphatase pair interacts with an ion channel to regulate ABA signaling in plant guard cells. Proc. Natl. Acad. Sci. U.S.A. 106, 21419–21424 (2009). 19955427 10.1073/pnas.0910601106 PMC2795491

[15] Y. Takahashi, Y. Ebisu, T. Kinoshita, M. Doi, E. Okuma, Y. Murata, K.-I. Shimazaki, bHLH transcription factors that facilitate K^+^ uptake during stomatal opening are repressed by abscisic acid through phosphorylation. Sci. Signal. 6, ra48 (2013). 23779086 10.1126/scisignal.2003760

[16] L. J. Pillitteri, K. U. Torii, Mechanisms of stomatal development. Annu. Rev. Plant Biol. 63, 591–614 (2012). 22404473 10.1146/annurev-arplant-042811-105451

[17] O. S. Lau, D. C. Bergmann, Stomatal development: A plant’s perspective on cell polarity, cell fate transitions and intercellular communication. Development 139, 3683–3692 (2012). 22991435 10.1242/dev.080523 PMC3445305

[18] S. K. Han, K. U. Torii, Lineage-specific stem cells, signals and asymmetries during stomatal development. Development 143, 1259–1270 (2016). 27095491 10.1242/dev.127712

[19] C. A. MacAlister, K. Ohashi-Ito, D. C. Bergmann, Transcription factor control of asymmetric cell divisions that establish the stomatal lineage. Nature 445, 537–540 (2007). 17183265 10.1038/nature05491

[20] O. S. Lau, K. A. Davies, J. Chang, J. Adrian, M. H. Rowe, C. E. Ballenger, D. C. Bergmann, Direct roles of SPEECHLESS in the specification of stomatal self-renewing cells. Science 345, 1605–1609 (2014). 25190717 10.1126/science.1256888 PMC4390554

[21] T. W. Kim, M. Michniewicz, D. C. Bergmann, Z. Y. Wang, Brassinosteroid regulates stomatal development by GSK3-mediated inhibition of a MAPK pathway. Nature 482, 419–422 (2012). 22307275 10.1038/nature10794 PMC3292258

[22] G. E. Gudesblat, J. Schneider-Pizoń, C. Betti, J. Mayerhofer, I. Vanhoutte, W. van Dongen, S. Boeren, M. Zhiponova, S. de Vries, C. Jonak, E. Russinova, SPEECHLESS integrates brassinosteroid and stomata signalling pathways. Nat. Cell Biol. 14, 548–554 (2012). 22466366 10.1038/ncb2471

[23] S. Wang, Z. Zhou, R. Rahiman, G. S. Y. Lee, Y. K. Yeo, X. Yang, O. S. Lau, Light regulates stomatal development by modulating paracrine signaling from inner tissues. Nat. Commun. 12, 3403 (2021). 34099707 10.1038/s41467-021-23728-2 PMC8184810

[24] O. S. Lau, Z. Song, Z. Zhou, K. A. Davies, J. Chang, X. Yang, S. Wang, D. Lucyshyn, I. H. Z. Tay, P. A. Wigge, D. C. Bergmann, Direct control of *SPEECHLESS* by PIF4 in the high-temperature response of stomatal development. Curr. Biol. 28, 1273–1280.e3 (2018). 29628371 10.1016/j.cub.2018.02.054 PMC5931714

[25] D. Samakovli, T. Tichá, T. Vavrdová, M. Ovečka, I. Luptovčiak, V. Zapletalová, A. Kuchařová, P. Křenek, Y. Krasylenko, T. Margaritopoulou, L. Roka, D. Milioni, G. Komis, P. Hatzopoulos, J. Šamaj, YODA-HSP90 module regulates phosphorylation-dependent inactivation of SPEECHLESS to control stomatal development under acute heat stress in *Arabidopsis*. Mol. Plant 13, 612–633 (2020). 31935463 10.1016/j.molp.2020.01.001

[26] G. R. Lampard, C. A. MacAlister, D. C. Bergmann, *Arabidopsis* stomatal initiation is controlled by MAPK-mediated regulation of the bHLH SPEECHLESS. Science 322, 1113–1116 (2008). 19008449 10.1126/science.1162263

[27] C. Han, Y. Liu, W. Shi, Y. Qiao, L. Wang, Y. Tian, M. Fan, Z. Deng, O. S. Lau, G. De Jaeger, M.-Y. Bai, KIN10 promotes stomatal development through stabilization of the SPEECHLESS transcription factor. Nat. Commun. 11, 4214 (2020). 32843632 10.1038/s41467-020-18048-w PMC7447634

[28] C. Bian, X. Guo, Y. Zhang, L. Wang, T. Xu, A. DeLong, J. Dong, Protein phosphatase 2A promotes stomatal development by stabilizing SPEECHLESS in *Arabidopsis*. Proc. Natl. Acad. Sci. U.S.A. 117, 13127–13137 (2020). 32434921 10.1073/pnas.1912075117 PMC7293623

[29] C. Chater, K. Peng, M. Movahedi, J. A. Dunn, H. J. Walker, Y.-K. Liang, D. H. McLachlan, S. Casson, J. C. Isner, I. Wilson, S. J. Neill, R. Hedrich, J. E. Gray, A. M. Hetherington, Elevated CO_2_-induced responses in stomata require ABA and ABA signaling. Curr. Biol. 25, 2709–2716 (2015). 26455301 10.1016/j.cub.2015.09.013 PMC4612465

[30] P. Jalakas, E. Merilo, H. Kollist, M. Brosche, ABA-mediated regulation of stomatal density is OST1-independent. Plant Direct 2, e00082 (2018). 31245747 10.1002/pld3.82 PMC6508810

[31] K. Ohashi-Ito, D. C. Bergmann, *Arabidopsis* FAMA controls the final proliferation/differentiation switch during stomatal development. Plant Cell 18, 2493–2505 (2006). 17088607 10.1105/tpc.106.046136 PMC1626605

[32] Z. Cai, J. Liu, H. Wang, C. Yang, Y. Chen, Y. Li, S. Pan, R. Dong, G. Tang, D. Barajas-Lopez Jde, H. Fujii, X. Wang, GSK3-like kinases positively modulate abscisic acid signaling through phosphorylating subgroup III SnRK2s in Arabidopsis. Proc. Natl. Acad. Sci. U.S.A. 111, 9651–9656 (2014). 24928519 10.1073/pnas.1316717111 PMC4084465

[33] T. Umezawa, N. Sugiyama, F. Takahashi, J. C. Anderson, Y. Ishihama, S. C. Peck, K. Shinozaki, Genetics and phosphoproteomics reveal a protein phosphorylation network in the abscisic acid signaling pathway in *Arabidopsis thaliana*. Sci. Signal. 6, rs8 (2013). 23572148 10.1126/scisignal.2003509

[34] P. Wang, L. Xue, G. Batelli, S. Lee, Y.-J. Hou, M. van Oosten, H. Zhang, W. A. Tao, J.-K. Zhu, Quantitative phosphoproteomics identifies SnRK2 protein kinase substrates and reveals the effectors of abscisic acid action. Proc. Natl. Acad. Sci. U.S.A. 110, 11205–11210 (2013). 23776212 10.1073/pnas.1308974110 PMC3703982

[35] L. J. Pillitteri, D. B. Sloan, N. L. Bogenschutz, K. U. Torii, Termination of asymmetric cell division and differentiation of stomata. Nature 445, 501–505 (2007). 17183267 10.1038/nature05467

[36] J. A. Lake, F. I. Woodward, Response of stomatal numbers to CO_2_ and humidity: Control by transpiration rate and abscisic acid. New Phytol. 179, 397–404 (2008). 19086289 10.1111/j.1469-8137.2008.02485.x

[37] A. Arif, J. Jia, B. Willard, X. Li, P. L. Fox, Multisite phosphorylation of S6K1 directs a kinase phospho-code that determines substrate selection. Mol. Cell 73, 446–457.e6 (2019). 30612880 10.1016/j.molcel.2018.11.017 PMC6415305

[38] B. Wang, A. N. Kettenbach, X. Zhou, J. J. Loros, J. C. Dunlap, The phospho-code determining circadian feedback loop closure and output in neurospora. Mol. Cell 74, 771–784.e3 (2019). 30954403 10.1016/j.molcel.2019.03.003 PMC6583785

[39] A. Perraki, T. A. DeFalco, P. Derbyshire, J. Avila, D. Séré, J. Sklenar, X. Qi, L. Stransfeld, B. Schwessinger, Y. Kadota, A. P. Macho, S. Jiang, D. Couto, K. U. Torii, F. L. H. Menke, C. Zipfel, Phosphocode-dependent functional dichotomy of a common co-receptor in plant signalling. Nature 561, 248–252 (2018). 30177827 10.1038/s41586-018-0471-x PMC6250601

[40] C. H. Park, Y. Bi, J.-H. Youn, S.-H. Kim, J.-G. Kim, N. Y. Xu, R. Shrestha, A. L. Burlingame, S.-L. Xu, M. B. Mudgett, S.-K. Kim, T.-W. Kim, Z.-Y. Wang, Deconvoluting signals downstream of growth and immune receptor kinases by phosphocodes of the BSU1 family phosphatases. Nat. Plants 8, 646–655 (2022). 35697730 10.1038/s41477-022-01167-1 PMC9663168

[41] A. Houbaert, C. Zhang, M. Tiwari, K. Wang, A. de Marcos Serrano, D. V. Savatin, M. J. Urs, M. K. Zhiponova, G. E. Gudesblat, I. Vanhoutte, D. Eeckhout, S. Boeren, M. Karimi, C. Betti, T. Jacobs, C. Fenoll, M. Mena, S. de Vries, G. De Jaeger, E. Russinova, POLAR-guided signalling complex assembly and localization drive asymmetric cell division. Nature 563, 574–578 (2018). 30429609 10.1038/s41586-018-0714-x

[42] K.-Z. Yang, M. Jiang, M. Wang, S. Xue, L.-L. Zhu, H.-Z. Wang, J.-J. Zou, E.-K. Lee, F. Sack, J. le, Phosphorylation of Serine 186 of bHLH transcription factor SPEECHLESS promotes stomatal development in *Arabidopsis*. Mol. Plant 8, 783–795 (2015). 25680231 10.1016/j.molp.2014.12.014

[43] M. Boudsocq, H. Barbier-Brygoo, C. Lauriere, Identification of nine sucrose nonfermenting 1-related protein kinases 2 activated by hyperosmotic and saline stresses in *Arabidopsis thaliana*. J. Biol. Chem. 279, 41758–41766 (2004). 15292193 10.1074/jbc.M405259200

[44] R. Yoshida, T. Umezawa, T. Mizoguchi, S. Takahashi, F. Takahashi, K. Shinozaki, The regulatory domain of SRK2E/OST1/SnRK2.6 interacts with ABI1 and integrates abscisic acid (ABA) and osmotic stress signals controlling stomatal closure in Arabidopsis. J. Biol. Chem. 281, 5310–5318 (2006). 16365038 10.1074/jbc.M509820200

[45] H. Fujii, P. E. Verslues, J. K. Zhu, Arabidopsis decuple mutant reveals the importance of SnRK2 kinases in osmotic stress responses in vivo. Proc. Natl. Acad. Sci. U.S.A. 108, 1717–1722 (2011). 21220313 10.1073/pnas.1018367108 PMC3029766

[46] Y. Takahashi, J. Zhang, P.-K. Hsu, P. H. O. Ceciliato, L. Zhang, G. Dubeaux, S. Munemasa, C. Ge, Y. Zhao, F. Hauser, J. I. Schroeder, MAP3Kinase-dependent SnRK2-kinase activation is required for abscisic acid signal transduction and rapid osmotic stress response. Nat. Commun. 11, 12 (2020). 31896774 10.1038/s41467-019-13875-y PMC6940395

[47] Z. Lin, Y. Li, Z. Zhang, X. Liu, C.-C. Hsu, Y. du, T. Sang, C. Zhu, Y. Wang, V. Satheesh, P. Pratibha, Y. Zhao, C.-P. Song, W. A. Tao, J.-K. Zhu, P. Wang, A RAF-SnRK2 kinase cascade mediates early osmotic stress signaling in higher plants. Nat. Commun. 11, 613 (2020). 32001690 10.1038/s41467-020-14477-9 PMC6992735

[48] F. Soma, F. Takahashi, T. Suzuki, K. Shinozaki, K. Yamaguchi-Shinozaki, Plant Raf-like kinases regulate the mRNA population upstream of ABA-unresponsive SnRK2 kinases under drought stress. Nat. Commun. 11, 1373 (2020). 32170072 10.1038/s41467-020-15239-3 PMC7069986

[49] A. Kumari, P. K. Jewaria, D. C. Bergmann, T. Kakimoto, Arabidopsis reduces growth under osmotic stress by decreasing SPEECHLESS protein. Plant Cell Physiol. 55, 2037–2046 (2014). 25381317 10.1093/pcp/pcu159 PMC4318929

[50] Y. Ding, H. Li, X. Zhang, Q. Xie, Z. Gong, S. Yang, OST1 kinase modulates freezing tolerance by enhancing ICE1 stability in *Arabidopsis*. Dev. Cell 32, 278–289 (2015). 25669882 10.1016/j.devcel.2014.12.023

[51] V. Chinnusamy, M. Ohta, S. Kanrar, B. H. Lee, X. Hong, M. Agarwal, J. K. Zhu, ICE1: A regulator of cold-induced transcriptome and freezing tolerance in *Arabidopsis*. Genes Dev. 17, 1043–1054 (2003). 12672693 10.1101/gad.1077503 PMC196034

[52] M. M. Kanaoka, L. J. Pillitteri, H. Fujii, Y. Yoshida, N. L. Bogenschutz, J. Takabayashi, J.-K. Zhu, K. U. Torii, *SCREAM/ICE1* and *SCREAM2* specify three cell-state transitional steps leading to *Arabidopsis* stomatal differentiation. Plant Cell 20, 1775–1785 (2008). 18641265 10.1105/tpc.108.060848 PMC2518248

[53] H. Kim, S.-H. Kim, D. H. Seo, S. Chung, S.-W. Kim, J.-S. Lee, W. T. Kim, J.-H. Lee, ABA-HYPERSENSITIVE BTB/POZ PROTEIN 1 functions as a negative regulator in ABA-mediated inhibition of germination in *Arabidopsis*. Plant Mol. Biol. 90, 303–315 (2016). 26667153 10.1007/s11103-015-0418-7

[54] B. K. Nelson, X. Cai, A. Nebenfuhr, A multicolored set of *in vivo* organelle markers for co-localization studies in Arabidopsis and other plants. Plant J. 51, 1126–1136 (2007). 17666025 10.1111/j.1365-313X.2007.03212.x

[55] M. Kubo, M. Udagawa, N. Nishikubo, G. Horiguchi, M. Yamaguchi, J. Ito, T. Mimura, H. Fukuda, T. Demura, Transcription switches for protoxylem and metaxylem vessel formation. Genes Dev. 19, 1855–1860 (2005). 16103214 10.1101/gad.1331305 PMC1186185

[56] T. Nakagawa, S. Nakamura, K. Tanaka, M. Kawamukai, T. Suzuki, K. Nakamura, T. Kimura, S. Ishiguro, Development of R4 gateway binary vectors (R4pGWB) enabling high-throughput promoter swapping for plant research. Biosci. Biotechnol. Biochem. 72, 624–629 (2008). 18256458 10.1271/bbb.70678

[57] T. Nakagawa, T. Kurose, T. Hino, K. Tanaka, M. Kawamukai, Y. Niwa, K. Toyooka, K. Matsuoka, T. Jinbo, T. Kimura, Development of series of gateway binary vectors, pGWBs, for realizing efficient construction of fusion genes for plant transformation. J. Biosci. Bioeng. 104, 34–41 (2007). 17697981 10.1263/jbb.104.34

[58] S. J. Clough, Floral dip: Agrobacterium-mediated germ line transformation. Methods Mol. Biol. 286, 91–102 (2005). 15310915 10.1385/1-59259-827-7:091

[59] S. Kumar, G. Stecher, K. Tamura, MEGA7: Molecular evolutionary genetics analysis version 7.0 for bigger datasets. Mol. Biol. Evol. 33, 1870–1874 (2016). 27004904 10.1093/molbev/msw054 PMC8210823

