# Supplementary material for: Abscisic acid regulates stomatal production by imprinting a SnRK2 kinase–mediated phosphocode on the master regulator SPEECHLESS
Source: Sci Adv. 2022 Oct 7;8(40):eadd2063. doi: 10.1126/sciadv.add2063 (PMC9544323; doi:10.1126/sciadv.add2063)
Supplement: Supplementary file 1 — Figs. S1 to S12 Table S1 [file sciadv.add2063_sm.pdf]

Science Advances
AAAS

Supplementary Materials for

Abscisic acid regulates stomatal production by imprinting an SnRK2 kinase–mediated phosphocode on the master regulator SPEECHLESS

Xin Yang *et al.*

Corresponding author: On Sun Lau, onsunlau@nus.edu.sg

Sci. Adv. **8**, eadd2063 (2022)
DOI: 10.1126/sciadv.add2063

This PDF file includes:

Figs. S1 to S12
Table S1

Supplementary Materials for

Abscisic acid regulates stomatal production by imprinting a SnRK2 kinase-mediated phosphocode on the master regulator SPEECHLESS

Xin Yang, Lalitha Gavya S, Zimin Zhou, Daisuke Urano, On Sun Lau*.

*Correspondence to: onsunlau@nus.edu.sg

This PDF file includes:

Figs. S1 to S12
Tables S1

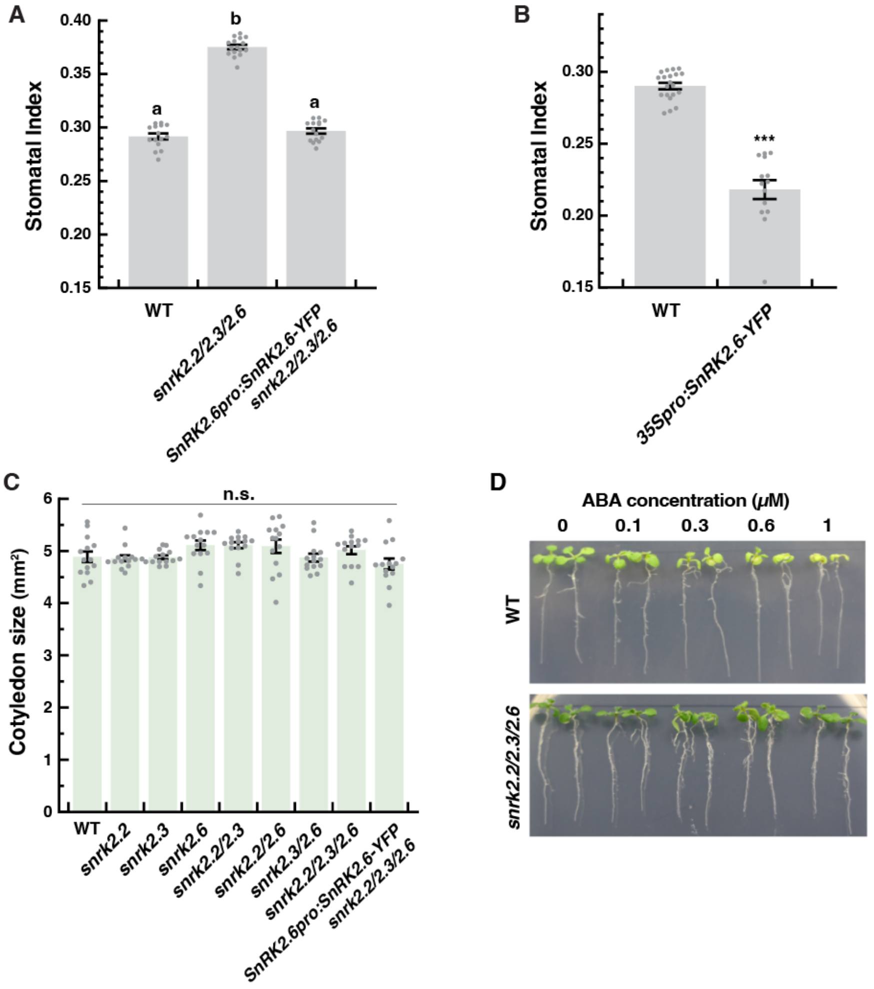

Fig. S1. Stomatal indices of *SnRK2*.6 complementing and overexpressed lines and growth characteristics of *snrk2* mutants.

(**A**) Quantification of stomatal indices of 10-day-old abaxial cotyledons of wild-type (WT), the triple *snrk2.2/2.3/2.6* mutant and *SnRK2.6pro:SnRK2.6-YFP* in *snrk2.2/2.3/2.6*. Data from WT is the same as in Fig. 2M since the experiments were conducted in parallel. The full rescue by the *SnRK2.6* transgene may be due to multiple copies of the transgene which would result in higher levels of *SnRK2.6* transcripts than WT. (**B**) Quantification of stomatal indices of 10-day-old abaxial cotyledons of wild-type (WT) and *35Spro:SnRK2.6-YFP*. (**C**) Quantification of cotyledon size of 10-day-old seedlings of the indicated genotypes. (**D**) Effect of ABA on the seedling growth of WT and the *snrk2* triple mutant. Seedlings were grown for 3 days and were transferred to ABA-containing media for 7 more days. Values are mean +/- SEM, $n \geq 14$. One-way ANOVA with Tukey's multiple comparisons test (A & C) or Student's t-test (B), $p<0.0001$, n.s.: not significant.

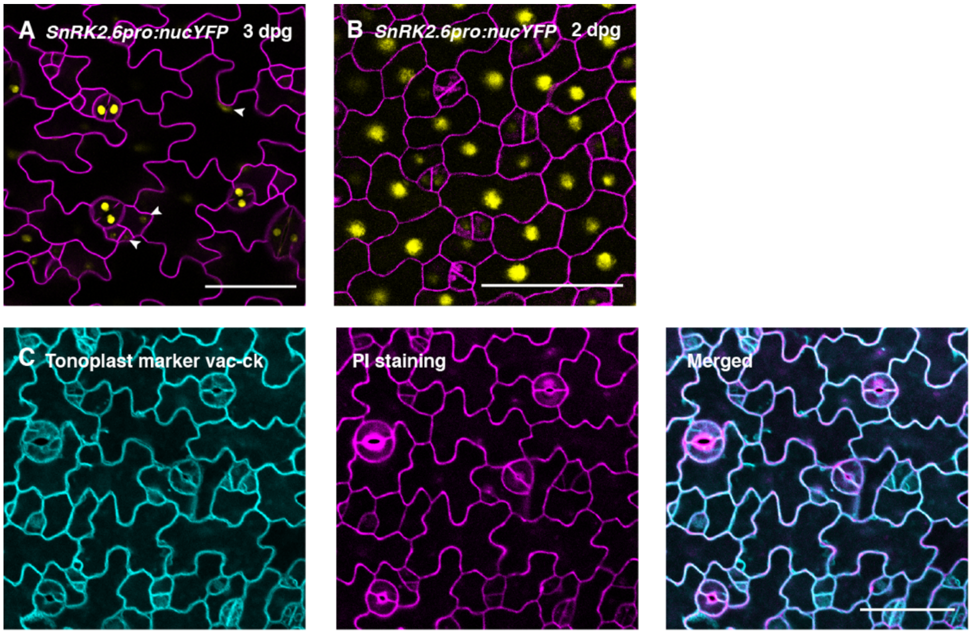

Fig. S2. Confocal analyses of a transcriptional reporter of *SnRK2.6* and a tonoplast reporter line.

(**A** & **B**) Confocal images of abaxial cotyledons of *SnRK2.6pro:nucYFP* examined at 3 (A) or 2 (B) days post-germination (dpg). For (A), arrowheads mark select early stomatal lineage and pavement cells having weak but detectable YFP signal. (**C**) Confocal images of 3-dpg abaxial cotyledons of a tonoplast reporter line, vac-ck (see Methods). Cell outlines were marked by *ML1pro:mCherry-RCI2A* (A & B) or propidium iodide (C) (magenta). Scale bar, 50 μm.

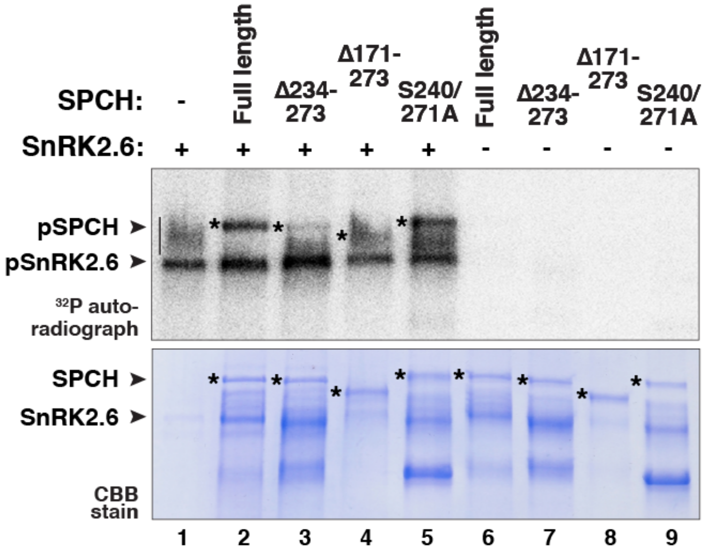

Fig. S3. ***In vitro*** **phosphorylation of SPCH by SnRK2.6 (independent replicate of Fig. 4B).**
Recombinant SPCH and its truncated/mutated variants were incubated alone or with recombinant SnRK2.6 in the presence of ^{32}P-labeled ATP. Upper panel: Autoradiograph of the ^{32}P-labeled proteins. Bottom panel: Coomassie blue (CBB) protein staining of total proteins. |: Probable phosphorylated species (non-specific) in the absence of substrate.*: full-length or truncated/mutated forms of SPCH proteins.

A Identification of a phosphopeptide from SnRK2.6-treated recombinant SPCH (WT)

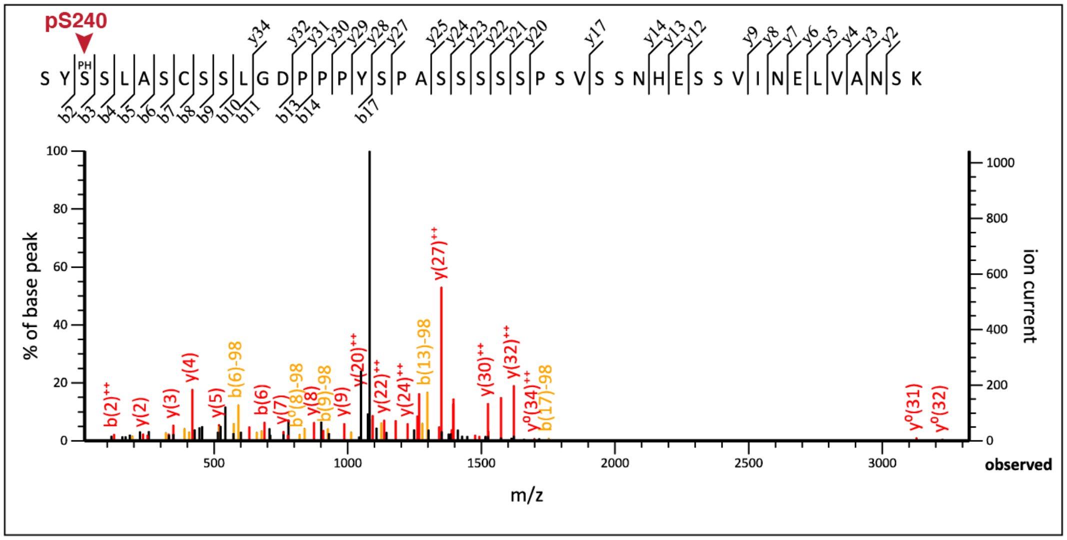

B Identification of a phosphopeptide from SnRK2.6-treated recombinant SPCH-S240/271A

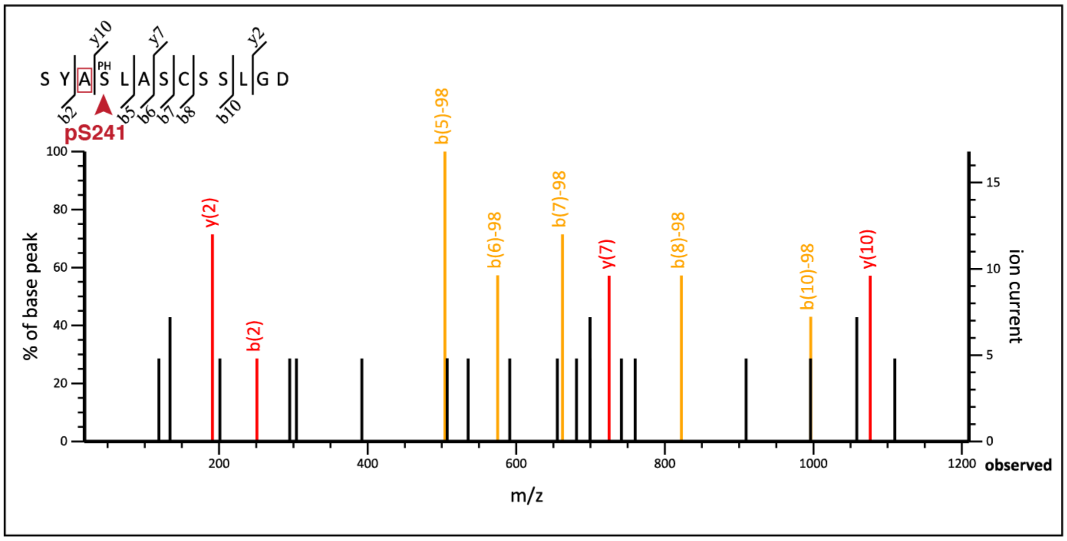

Fig. S4. Mass spectrometry identification of SPCH residues phosphorylated by SnRK2.6. *In vitro* phosphorylation assays on recombinant SPCH (WT version) or SPCH-S240/271A by SnRK2.6 were carried out and the SPCH proteins were analyzed by LC-MS/MS (see Methods). (**A**) MS/MS spectra of a SPCH phosphopeptide S238-K282, phosphorylated at S240 (arrowhead), derived from WT SPCH. (**B**) MS/MS spectra of a phosphopeptide S238-D250, derived from the mutated SPCH-S240A/S271A protein. Arrowhead denotes the phosphorylation site at S241. Note the amino acid substitution from serine to alanine at 240 (red bracket) in the mutated variant. Red peaks are fragments assigned to b or y ions, and yellow peaks are neutral loss (-H_3PO_4) fragments.

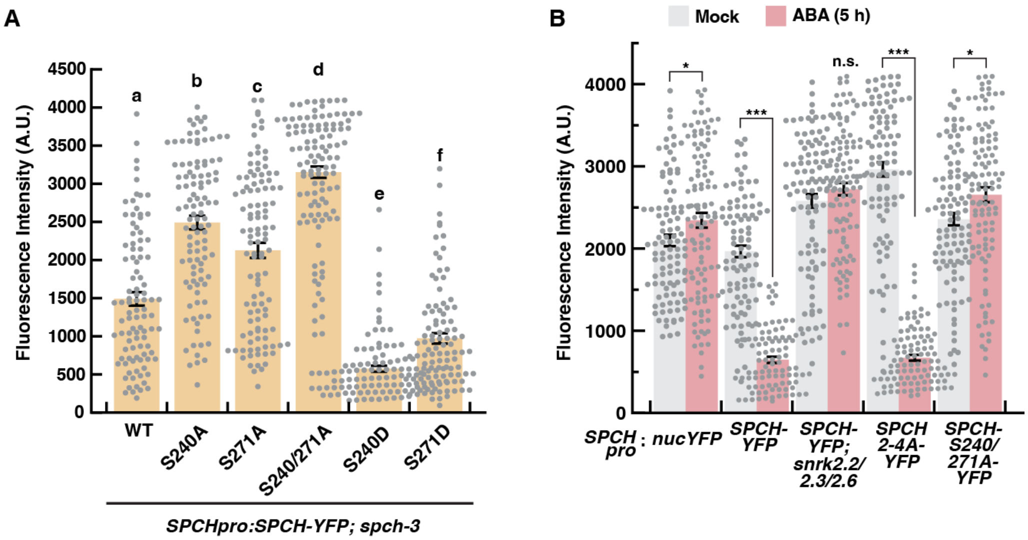

Fig. S5. Quantification of the fluorescence signal intensity of *SPCH* reporter lines.

YFP fluorescence signals from the nuclei of confocal images related to Fig. 4K (A) and Fig. 5K (B) were quantified by ImageJ. Values are mean +/- SEM, n ≥ 94 nuclei. One-way ANOVA with Tukey's multiple comparisons test, $p<0.05$ (A). Student's t-test, *, $p<0.05$, ***, $p<0.001$ (B).

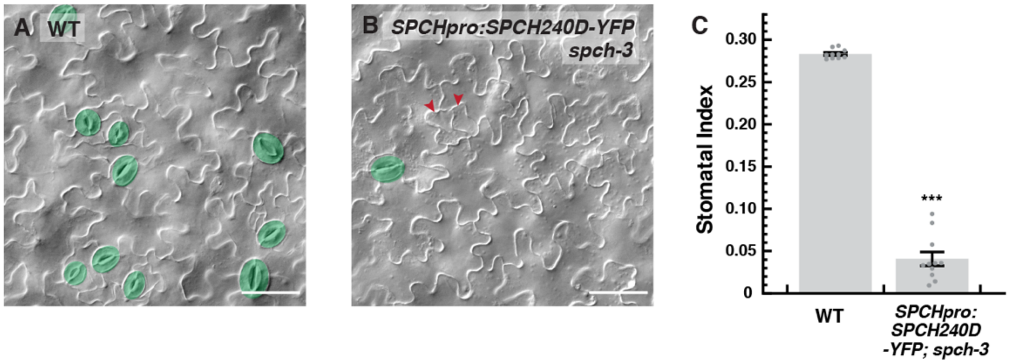

Fig. S6. Partial rescue of *spch* mutant by *SPCHpro:SPCH240D-YFP*.

Representative images (A & B) and quantification of stomatal indices (C) of 10-day-old abaxial cotyledons of wild-type (WT; A) and *SPCHpro:SPCH240D-YFP* in *spch-3* (B). Stomatal number was substantially lower in the latter line, and likely aborted stomatal lineage cells (arrowheads) were observed occasionally. Scale bar: 50 μm. Values are mean +/- SEM, n ≥ 10. Student's t-test, p<0.0001.

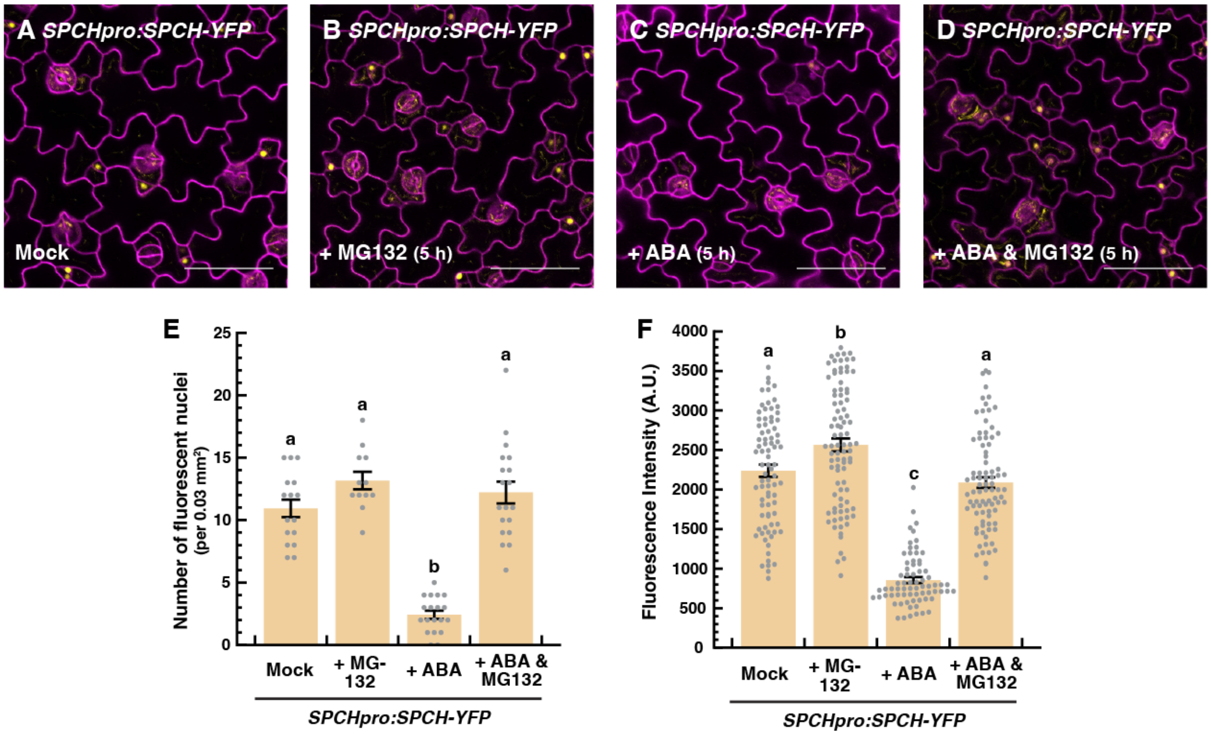

Fig. S7. Effect of the proteasome inhibitor MG132 on the ABA-induced suppression of SPCH.

Confocal images of 4-day-old abaxial cotyledons of *SPCHpro:SPCH-YFP* treated with mock (A), 20 µM MG132 (B), 60 µM ABA (C), and 60 µM ABA plus 20 µM MG132 (D) for 5 h. Images were taken with the same excitation and acquisition settings. The number of YFP-expressing cells (yellow) (E) and the YFP fluorescence intensity of the nuclei (F) were quantified. Values are mean +/- SEM, n ≥ 12 independent cotyledons (E) or 70 independent nuclei (F). One-way ANOVA with Tukey's multiple comparisons test, p<0.01. Cell outlines were visualized with propidium iodide (magenta). Scale bar, 50 µm.

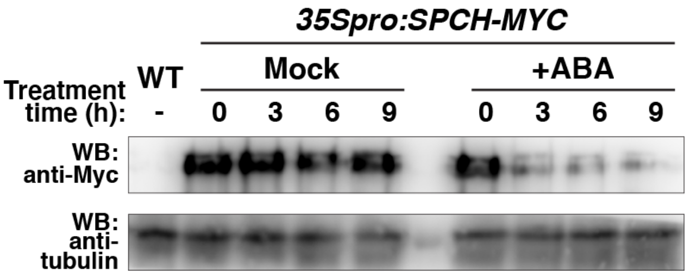

Fig. S8. Time-course immunoblot analysis of *35Spro:SPCH-MYC* in response to ABA treatment.

Three-day-old seedlings of *35Spro:SPCH-MYC* were transferred to media without (Mock) or with 60 μM ABA. Seedlings were harvested at the indicated time points, and total soluble proteins were used for immunoblot analyses with anti-Myc or anti-tubulin antibodies.

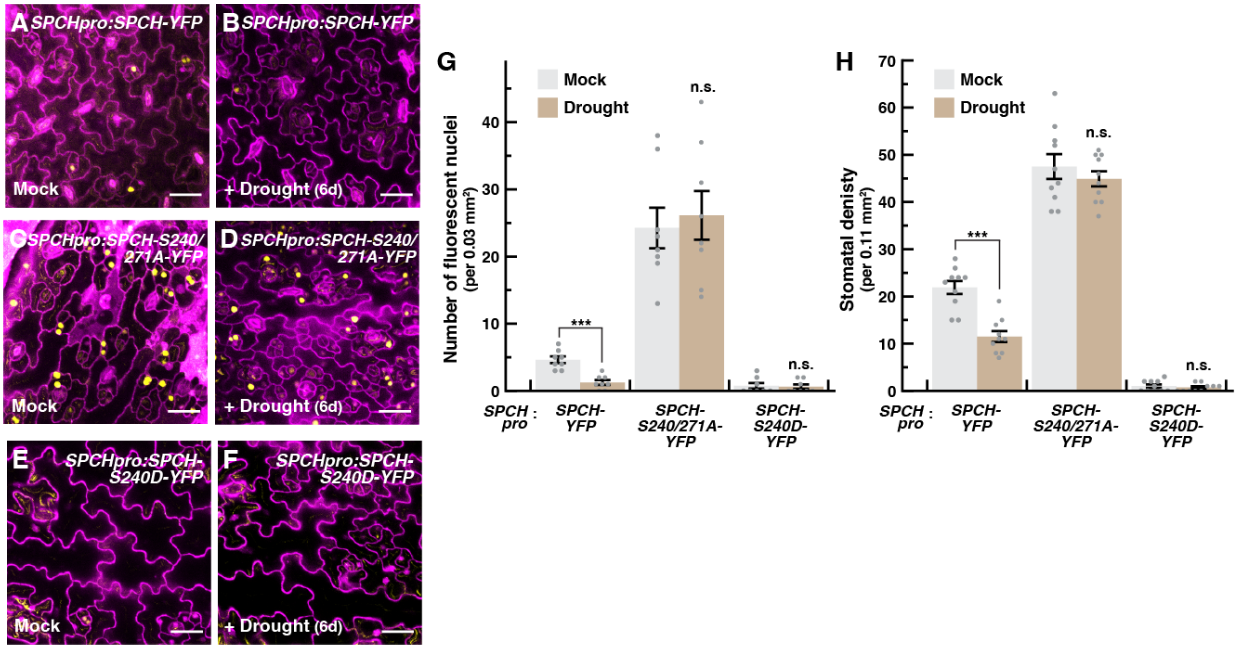

Fig. S9. Effect of drought on SPCH levels and stomatal production in the adult Arabidopsis plants expressing SPCH-YFP or its variants.

(**A**-**G**) Four-week-old Arabidopsis of *SPCHpro:SPCH-YFP* (A & B), *SPCHpro:SPCH-S240/271A-YFP* (C & D) and *SPCHpro:SPCH-S240D-YFP* (E & F) (all in *spch-3* background) were treated with mock (A, C & E) or drought (withholding water; B, D & F). At 6 days post-treatment, images of the abaxial surface of young leaves emerged after the start of the drought treatment (see Methods) were taken with the same excitation and acquisition settings. The numbers of YFP-expressing cells (yellow) are quantified (G). (**H**) Stomatal density of the respective *SPCH* lines at Day 9 after the start of the drought treatment. Data were derived from DIC images. Values are mean +/- SEM, n = 8 (G) or 10 (H) independent leaves. Student's t-test, $p<0.01$. Cell outlines were visualized with propidium iodide (magenta). Scale bar, 25 μm.

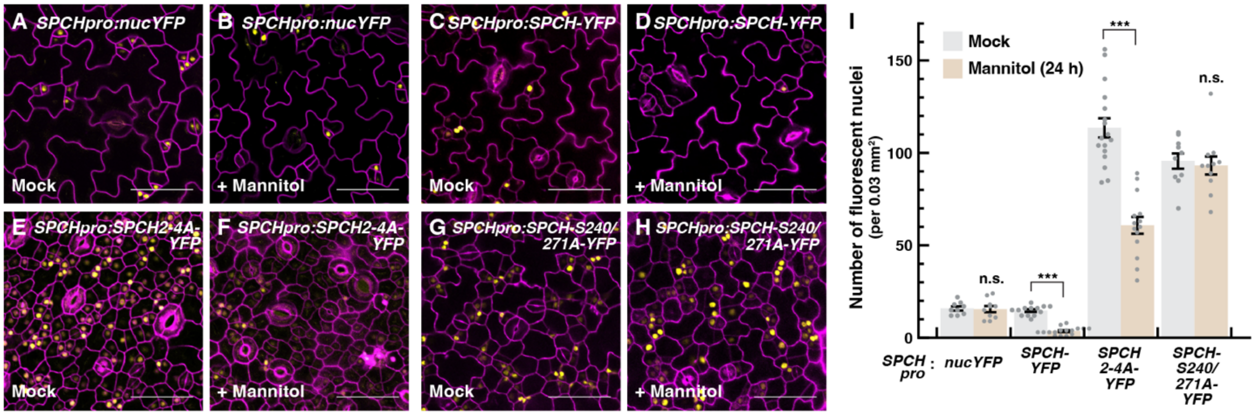

Fig. S10. SnRK2-targeted residues on SPCH transduce osmotic stress-mediated suppression of SPCH. (**A** to **I**) Effect of osmotic stress on the transcriptional and translational reporters of *SPCH* and its phospho variants. Three-day old seedlings of *SPCHpro:nucYFP* (A & B), *SPCHpro:SPCH-YFP* (C & D), *SPCHpro:SPCH2-4A-YFP* (E & F) and *SPCHpro:SPCH-S240/271A-YFP* (G & H) when treated with mock (A, C, E & G) or mannitol (200 mM; B, D, F & H) for 24 h (all translational reporters were in *spch-3* background). Images of the abaxial cotyledons were taken with the same excitation and acquisition settings. The numbers of YFP-expressing cells (yellow) are quantified (I). Values are mean +/- SEM, n ≥ 9 independent cotyledons. Student's t-test, $p<0.01$. Cell outlines were visualized with propidium iodide (magenta). Scale bar, 50 µm.

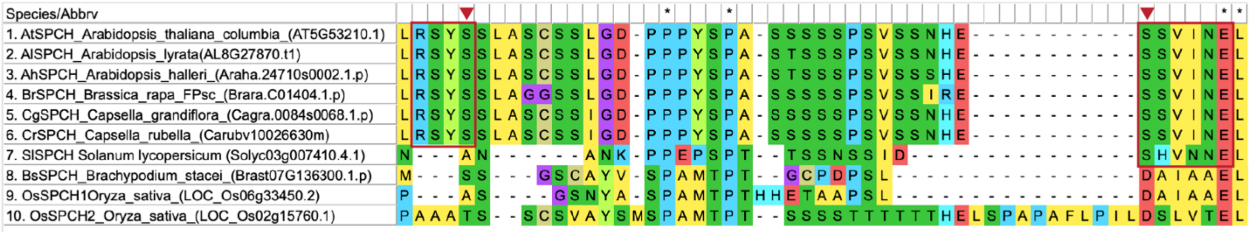

Fig. S11. Sequence alignment of the SnRK2-targeted MPKTD region of Arabidopsis SPCH and its homologues from other plant species.

The putative SnRK2 target motifs, RxxS and SxxxxE, were marked by brackets and the position of S240 and S271 of Arabidopsis SPCH are marked by triangles. Sequences were retrieved from Phytozome (https://phytozome-next.jgi.doe.gov).

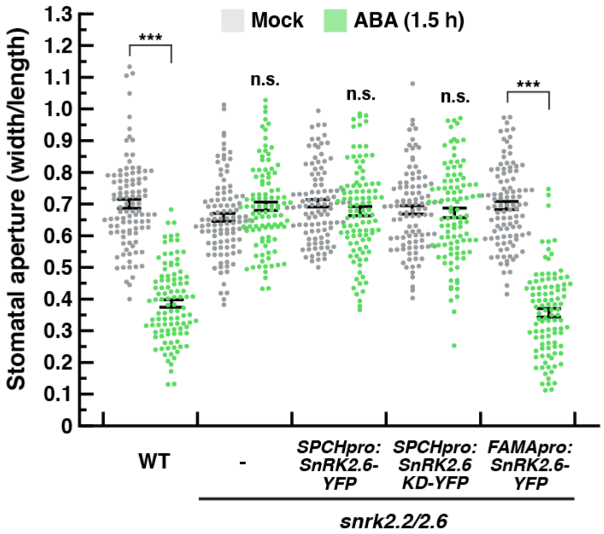

Fig. S12. Defect in ABA-induced stomatal closure of *snrk2* mutants can be rescued by expressing *SnRK2.6* at late, but not early, stage of stomatal development.

To assay for the changes in stomatal aperture triggered by ABA, seedlings (5 dpg) of WT, *snrk2.2/2.6*, and *snrk2.2/2.*6 transformed with either *SPCHpro:SnRK2.6-YFP*, *SPCHpro:SnRK2.6KD-YFP* or *FAMApro:SnRK2.6-YFP* (see text) were incubated with stomatal opening buffer before the addition of mock solution or ABA (10 µM) for 1.5 h (see Methods). Data from WT are the same as in Fig. 6B as the experiments were conducted in parallel. Stomatal aperture is represented as the inner width to length ratio of each stoma. Values are mean +/- SEM, n ≥ 100 stomata. Student's t-test, $p<0.01$.

Table S1. List of primers used for site-directed mutagenesis.

Gene name	Mutation	Forward primer	Reverse primer
SnRK2.6	K50N	GAGCTTGTTGCTGTTAATTATATCGAG AGAGGTG	CACCTCTCTCGATATAATTAACAGCAA CAAGCTC
SPCH	S240A	CCGCTTCGCTCTTACGCCTCATTGGC CAGTTG	CAACTGGCCAATGAGGCGTAAGAGCG AAGCGG
SPCH	S240D	CGCTTCGCTCTTACGACTCATTGGCC AGTTG	CAACTGGCCAATGAGTCGTAAGAGCG AAGCG
SPCH	S271A	GTTAGTAGTAACCATGAGGCTAGTGT GATCAATGAGC	GCTCATTGATCACACTAGCCTCATGGT TACTACTAAC
SPCH	S271D	GTTAGTAGTAACCATGAGGATAGTGT GATCAATGAGC	GCTCATTGATCACACTATCCTCATGGT TACTACTAAC

* All sequences are from 5’ to 3’ orientation.
